# Supplementary material for: Physical activity, immune function and inflammation in kidney patients (the PINK study): a feasibility trial protocol
Source: BMJ Open. 2017 May 29;7(5):e014713. doi: 10.1136/bmjopen-2016-014713 (PMC5729975; doi:10.1136/bmjopen-2016-014713)
Supplement: Supplementary material 1 [file bmjopen-2016-014713supp001.pdf]

Participant Code :

Date :

Tiredness Scale

Place a mark on the horizontal line below after considering how you feel:

Generally over the last few days, how tired have you felt?

I have felt overwhelmingly tired and feel like I can't do anything	|———————————————|	I haven't felt tired at all and I am full of energy

Over the last few days how tired have your muscles felt?

My muscles have felt very tired and heavy	|———————————————|	My muscles haven't felt tired at all

Over the last few days how out of breath have you felt?

I have felt very out of breath	|———————————————|	Not at all out of breath

Over the last few days how mentally tired or sleepy have you felt during the day?

I have felt very mentally tired or sleepy or have had trouble staying awake at times	|———————————————|	I've felt wide awake and alert all day haven't felt sleepy at all

In your own words, describe how "being tired" feels to you?
