# Supplementary material for: Physical activity, immune function and inflammation in kidney patients (the PINK study): a feasibility trial protocol
Source: BMJ Open. 2017 May 29;7(5):e014713. doi: 10.1136/bmjopen-2016-014713 (PMC5729975; doi:10.1136/bmjopen-2016-014713)
Supplement: Author's manuscript [file bmjopen-2016-014713.draft_revisions.pdf]

BMJ Open

Physical Activity, Immune Function and Inflammation in Kidney Patients : the PINK Study Protocol

Journal:	*BMJ Open*
Manuscript ID	bmjopen-2016-014713
Article Type:	Protocol
Date Submitted by the Author:	21-Oct-2016

Complete List of Authors:	Highton, Patrick; Loughborough University, School of Sport, Exercise and Health Sciences; University of Leicester, Department of Infection, Immunity and Inflammation<br>Neale, Jill; University of Leicester, Department of Infection, Immunity and Inflammation<br>Wilkinson, Thomas; University of Leicester, Department of Infection, Immunity and Inflammation<br>Bishop , Nicolette ; Loughborough University, School of Sport, Exercise and Health Sciences; University of Leicester, Department of Infection, Immunity and Inflammation<br>Smith, Alice; University of Leicester, Department of Infection, Immunity and Inflammation; Loughborough University, School of Sport, Exercise and Health Sciences

**Primary Subject Heading**:	Immunology (including allergy)
Secondary Subject Heading:	Sports and exercise medicine, Renal medicine
Keywords:	Renal transplantation < NEPHROLOGY, IMMUNOLOGY, Inflammation, Exercise

SCHOLARONE™
Manuscripts

Physical Activity, Immune Function and Inflammation in Kidney Patients: the PINK Study Protocol

Patrick J Highton[1,2], Jill Neale[2], Thomas J Wilkinson[2], Nicolette C Bishop[1,2], Alice C Smith[1,2].

Corresponding author:

Patrick J Highton
Leicester Kidney Exercise Team
Academic Unit
Leicester General Hospital
LE5 4PW
P.Highton@lboro.ac.uk
0116 258 4346

[1] School of Sport, Exercise and Health Sciences, Loughborough University, Loughborough, United Kingdom

[2] Department of Infection, Immunity and Inflammation, University of Leicester, Leicester, United Kingdom

Keywords: renal transplantation, nephrology, immunology, inflammation, exercise

Word count (excluding title page, abstract, references, figures and tables): 1930

For peer review only

For peer review only - http://bmjopen.bmj.com/site/about/guidelines.xhtml

Abstract

Introduction: Chronic Kidney Disease (CKD) patients display elevated cardiovascular risk only partly attributed to traditional risk factors, as well as increased infection-related mortality. CKD patients also exhibit a pro-inflammatory environment and impaired immune function. Aerobic exercise has the potential to positively impact these detriments, but is under-researched in this patient population. This feasibility study will investigate the effects of acute aerobic exercise on inflammation and immune function in CKD, to inform the design of larger studies intended to ultimately influence current exercise recommendations.

Methods and analysis: Patients with CKD, including renal transplant recipients, will visit the laboratory on two occasions, both preceded by appropriate exercise, alcohol and caffeine restrictions. On Visit 1, baseline assessments will be completed, comprising anthropometrics, body composition, cardiovascular function, and fatigue and leisure time exercise questionnaires. Participants will then undertake an incremental shuttle walk test, to estimate VO_2peak. On Visit 2, participants will complete a 20 minute shuttle walk at a constant speed to achieve 85% estimated VO_2peak. Blood and saliva samples will be taken before, immediately after and 1 hour after this exercise bout. Muscle O_2 saturation will be monitored throughout exercise and recovery. Age and sex-matched non-CKD 'healthy control' participants will complete an identical protocol. Blood and saliva samples will be analysed for markers of inflammation and immune function, using standard assay and flow cytometry techniques. Appropriate statistical tests will be used to analyse the data.

Ethics and dissemination: A favourable opinion was granted by the East Midlands-Derby Research Ethics Committee on 18/09/2015 (ref 15/EM/0391) and the study was approved and sponsored by University Hospitals of Leicester Research and Innovation (ref 11444). The study was registered with ISRCTN (ref 38935454). The results will be presented at relevant conferences, and it is anticipated that the reports will be published in appropriate journals in late 2017.

Strengths and limitations of this study
• Inclusion of control group matched for age and sex<br>• A variety of outcome measures to inform future study design<br>• Pragmatic study design which will inform future exercise recommendations for patients with CKD<br>• No non-exercise control visit

Introduction

Patients with all stages of Chronic Kidney Disease (CKD) have elevated cardiovascular disease (CVD) risk that cannot be fully explained by traditional risk factors (1). CVD is the most common cause of death amongst CKD patients (23% in 2013), followed by infection (19% in 2013) (2).

CKD patients are often sarcopenic and obese (3–6), and further deterioration in these characteristics is often observed after renal transplantation (7–9). Patients with CKD and renal transplant recipients (RTRs) also frequently suffer from chronic systemic inflammation (10,11), which can worsen cachexia and increase cardiovascular risk in CKD (12,13). CKD patients and RTRs also display impaired cellular immune function(14–16), which may explain why infection is the second-leading cause of death in this population (2). This effect (i.e. impaired immune function) is further compounded in RTRs by the immunosuppressive drug regime. Impaired immune function may

For peer review only - http://bmjopen.bmj.com/site/about/guidelines.xhtml

further exacerbate inflammation due to alterations in circulating immune cell subsets (17) which could serve to worsen body composition and increase cardiovascular risk.

Exercise has the potential to benefit and improve many of the aforementioned inter-related morbidities. In the general population, exercise can positively impact upon weight gain (18), muscle wasting (19), physical capacity (20) and fatigue (21). Further, moderate aerobic exercise can also modify systemic inflammation (22,23) and bolster immune function (24,25).

In the CKD population, similar positive effects of exercise on body composition, physical function and quality of life have been demonstrated (26–29), however the research on inflammation and immune function in this population is limited. Previous research has shown that 30 minutes of moderate intensity walking exercise elicited a normal pattern of leukocyte and monocyte activation, whilst enhancing granulocyte function and promoting an anti-inflammatory environment (increased Interleukin-10 release) in pre-dialysis CKD patients (30). However, in a similar population an exhaustive bout of cycling exercise elicited a shift towards the more pro-inflammatory $CD16^+$ monocyte, potentially favouring inflammation (31). This disparity may be due to the exercise intensities investigated – moderate intensity exercise is more beneficial in strengthening immune function and preventing infection (24) and is promoted in general exercise guidelines. As such, investigating the effects of moderate intensity exercise in this patient population is more pragmatic and will help to guide future exercise recommendations, which are lacking in this patient population.

Therefore, this feasibility study aims investigate the effects of acute aerobic walking exercise on the inflammation and immune function in CKD patients. This will create the basis from which a larger trial can be conducted, including data for a power calculation, which will ultimately help to inform exercise recommendations in this population. As such, no power calculation has been completed for this study.

Methods and Analysis

Outcomes to be measured

This study will investigate the effect of 20 minutes of moderate intensity walking exercise on immune cell subsets and inflammatory markers. Participants will be grouped based on their status (i.e. 'patient' or 'healthy control'). Both groups will complete an identical acute, cross-sectional study protocol necessitating two study visits, as explained below.

Participant recruitment

All CKD patients will be screened for eligibility by their own clinician prior to recruitment, and approached during their routine outpatient appointments. The inclusion and exclusion criteria for patients are summarised in Table 1. Healthy age and sex matched controls with no known chronic disease will be recruited from the local community. Those who do not believe themselves to suffer from any significant chronic disease will be eligible to participate.

For peer review only - http://bmjopen.bmj.com/site/about/guidelines.xhtml

Inclusion Criteria	Exclusion Criteria
▪ Established chronic kidney disease (all stages will be eligible including those with an established kidney transplant)	▪ Age under 18 years<br>▪ Pregnancy<br>▪ Received kidney transplant less than 6 months prior to study entry<br>▪ Any element of study assessment protocol considered by own clinician to be contraindicated due to physical impairment, comorbidity or any other reason<br>▪ Inability to give informed consent for any reason<br>▪ Visual or hearing impairment or insufficient command of English to give informed consent or comply with the assessment protocol

Table 1. Inclusion and exclusion criteria for patients.

Trial design and timeline

This is a non-randomised, controlled, feasibility study. Participants will complete two study visits as described below:

Visit 1: Participants will arrive at the laboratory in the morning, having consumed no caffeine or alcohol and completed no strenuous exercise for 24 hours. Participants will complete questionnaires about time spent in leisure activity and their perception of fatigue, and assessments of anthropometry, body composition and cardiovascular condition. Participants will then undertake the Incremental Shuttle Walk Test (ISWT), followed by the Endurance Shuttle Walk Test (ESWT) as explained below. If the participant cannot complete the full 20 minute duration of the ESWT, they will be withdrawn from the study.

Visit 2: Participants will arrive at the laboratory in the morning, following the same standardisation procedure as in Visit 1. After resting for 10 minutes, blood and saliva samples will be collected. The ESWT will then be completed at the same speed as in Visit 1, lasting for 20 minutes. Within 5 minutes of exercise cessation, another blood and saliva sample will be collected. The participant will then rest for 1 hour, after which a final blood and saliva sample will be collected. This protocol is summarised in a schematic in Figure 1, and the outcome measures are explained in greater detail below.

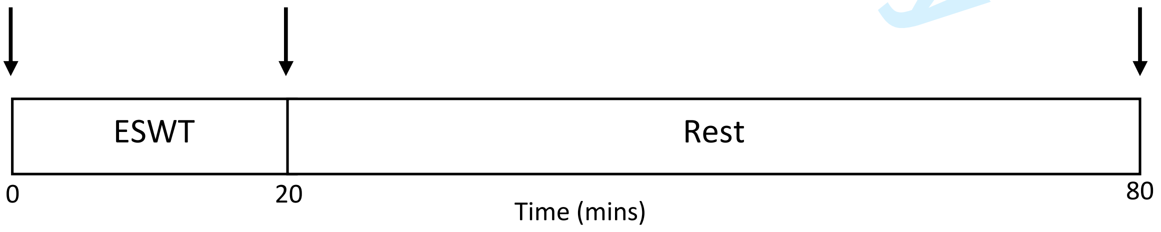

Figure 1. Visit 2 exercise and sample collection protocol. ↓= Venous blood and saliva collection. ESWT = Endurance Shuttle Walk Test

For peer review only - http://bmjopen.bmj.com/site/about/guidelines.xhtml

Physical performance

Endurance Capacity will be assessed using the ISWT (32) and ESWT (33). In the ISWT, the participant walks a level 10m shuttle course at a speed controlled by an external audible bleep signal. The test progressively increases at 1 minute intervals for a total of 12 intervals, and is terminated when the participant fails to complete a shuttle within the required time. Following the ISWT, a walking speed equating to 85% of predicted peak O_2 consumption (VO_2peak) can be calculated. The ESWT is completed at this continuous speed on the same shuttle course until volitional exhaustion or the end of the test (20 minutes) is reached.

Venous blood sampling

Venous blood will be collected using venepuncture of the antecubital vein of either arm – provided the absence of an arteriovenous fistula. Blood will be drawn through a 21-guage needle and collected into K_2EDTA (di-potassium ethylenediaminetetraacetic acid), sodium citrate, sodium heparin and blank monovettes.

Blood processing, storage and analysis

Blood collected into EDTA tubes will be centrifuged at 4°C; the supernatant will then be aliquoted and frozen at -80°C for future analysis of pro-inflammatory cytokines using Enzyme-Linked Immunosorbent Assay (ELISA). Blood collected into sodium citrate will be double-centrifuged at room temperature (15 mins at 2,500g, supernatant aliquoted followed by another 15 mins at 2,500g) to create platelet-free plasma; the supernatant will then be aliquoted and frozen at -80°C for future phenotyping of microparticles using flow cytometry. Blood collected into sodium heparin will be analysed on the day of collection for immune cell subsets using flow cytometry, as explained below. Blood collected into blank monovettes will be sent to the Diagnostic Pathology Service at University Hospitals of Leicester NHS Trust on the day of collection for renal profile analysis.

Flow cytometry

Immune cells will be characterised based on their expression of surface antigens using flow cytometry. Monocyte subsets will be categorised as classical ($CD14^{++}CD16^{-}$), intermediate ($CD14^{++}CD16^{+}$) and non-classical ($CD14^{+}CD16^{++}$) (34). Monocytic ACE expression will be assessed using CD143, with IgG1 as a negative control. T cells will be categorised as helper ($CD3^{+}CD4^{+}$) cytotoxic ($CD3^{+}CD8^{+}$) and regulatory ($CD4^{+}CD25^{+}CD127^{-}$) T cells. B Cells will be identified as $CD3^{+}CD19^{+}$ and NK Cells as CD3-$CD56^{+}$. Following appropriate staining and washing procedures, immune cells will be analysed on a FACS Calibur (BD Biosciences, Oxford, UK). Acquisition templates and preliminary flow cytometry results are displayed in Figure 2.

For peer review only - http://bmjopen.bmj.com/site/about/guidelines.xhtml

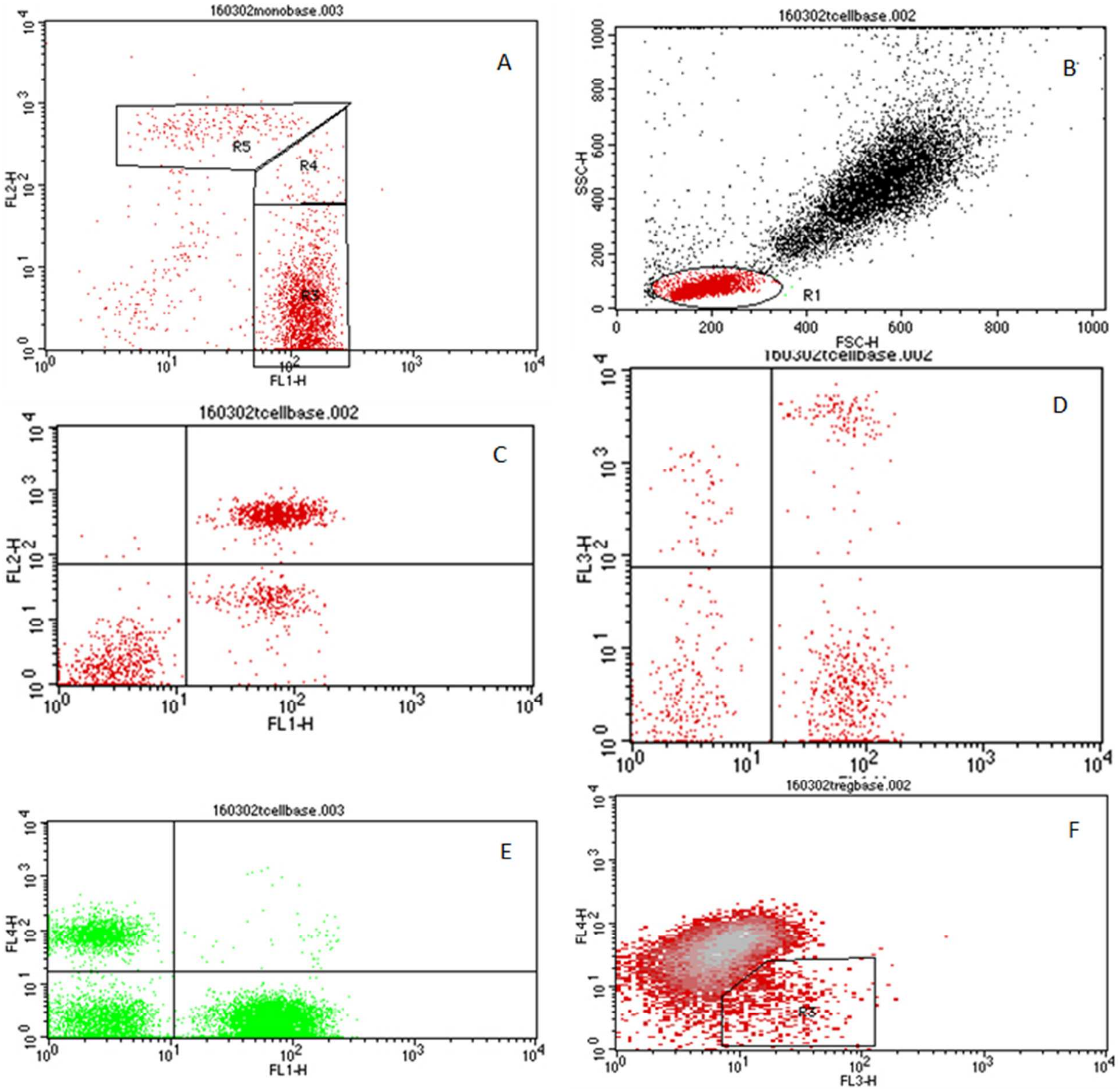

Figure 2. Demonstration of the gating strategy used to identify immune cell subsets. A: Monocyte subsets (R3 = classical ($CD14^{++}CD16$-), R4 = intermediate ($CD14^{++}CD16^{+}$), R5 = non-classical ($CD14^{+}CD16^{++}$) on a CD14 (FL1) vs CD16 (FL2) plot. B: lymphocyte gating from standard forward vs. side scatter plot. C: Gating $CD4^{+}$ helper T lymphocytes in the upper right quadrant, on a CD3 (FL1) vs CD4 (FL2) plot. D: Gating $CD8^{+}$ cytotoxic T lymphocytes in the upper right quadrant, on a CD3 (FL1) vs. CD8 (FL3) plot. E: Gating B lymphocytes in the upper left quadrant, on a CD3 (FL1) vs. CD19 (FL4) plot. F: Gating regulatory T cells on a CD25 (FL3) vs. CD127 (FL4) plot. Not all graphs display 100% of acquired cells – this has been altered independently to allow ease of gating.

For peer review only - http://bmjopen.bmj.com/site/about/guidelines.xhtml

Saliva sampling and storage

Saliva samples will be collected into sterile plastic containers. Participants will swallow to empty the mouth, then open and hold the container themselves before performing a passive dribble of saliva collected under the tongue over the next two minutes. Following centrifugation, the supernatant will be aliquoted and frozen for future analysis, primarily for secretory Immunoglobulin A (sIgA) to investigate mucosal immunity.

Anthropometric measures

Height, weight and waist and hip circumference will be measured using standard procedures (35).

Body composition

Body composition parameters (i.e. fat and fat free mass content) will be measured using bioelectrical impedance analysis (BIA) (Inbody 370, Chicago, IL, USA). BIA has been validated for use in CKD (36).

Cardiovascular condition and function

Cardiac bioreactance analysis will be carried out using a Non-Invasive Cardiac Output Monitor (NICOM) (Cheetah Medical, Maidenhead, UK). Cardiac bioreactance is a quick, safe, validated (37), and non-invasive method of assessing central haemodynamics including heart rate, stroke volume, cardiac output, and total peripheral resistance. This is accomplished by using cutaneous electrodes placed on the chest in combination with an automatic sphygmomanometer to measure blood pressure.

Time spent in leisure activities

The Leisure Time Exercise Questionnaire (LTEQ) assesses the amount of exercise a patient undertakes, from which a metabolic equivalent (METS) estimation can be calculated. It also identifies preferred physical activities.

Fatigue

Fatigue is a common complaint CKD, and it has multiple potential mechanisms including anaemia (38) and cachexia (39). To assess self-reported fatigue, participants will be asked to fill in a fatigue scale (an internally designed Likert scale), as well as the validated Functional Assessment of Chronic Illness Therapy-Fatigue (FACIT-F) (40) which has been extensively used in CKD populations (41)

Muscle O_2 saturation

Reduced muscle O_2 saturation is another possible physiological mechanism of fatigue (42) and may be related to increased inflammation (43). A small non-invasive muscle O_2 saturation device (BSXInsight, BSXAthletics, TX, USA) will be fitted around the participant's calf and worn during exercise bouts and subsequent recovery. This device uses near infrared spectroscopy (NIRS) to measure haemoglobin and myoglobin oxygenation (muscle O_2 tissue stores). NIRS has previously been used in a variety of disease conditions (44).

Clinical information

Clinical records will be used to extract information that allows associations to be made with physical activity levels and account for confounding variables, including: age; gender; ethnicity; primary cause of renal failure; transplant or dialysis type; time since transplant or duration of dialysis; co-morbidities; blood and urine test results; and current medications.

Data analysis plan

All outcome measures will be subject to the appropriate statistical test, based upon their position within the study design and their distribution. Data analysis will compare groups at each time point,

For peer review only - http://bmjopen.bmj.com/site/about/guidelines.xhtml

investigate any changes elicited by exercise, and any other pertinent outcomes. We will also explore interactions between variables, in order to generate hypotheses for future, larger studies.

Ethics and dissemination

Ethical and safety considerations

This protocol was reviewed by the East Midlands-Derby Research Ethics Committee (REC) and was given a favourable opinion (REC ref 15/EM/0391) on 24/09/2015. Additionally, local approval was given by the Research and Innovation office at the University Hospitals of Leicester NHS Trust (ref 11444) on 29/09/2015. Therefore, all steps have been taken when designing this protocol to minimise all ethical implications and ensure patient welfare. Two Substantial Amendments were made to this protocol, approved on 03/12/15 and 01/08/16 to add saliva collection, and fatigue questionnaires and muscle O_2 saturation respectively. The protocol presented here represents the most up to date version.

Dissemination plan

The results are planned for publication in late 2017/early 2018. We plan to present the data at relevant national and international conferences, as well as publish the findings in relevant journals. Participant level data will be available at a later date.

Trial registration

This study is registered with the ISRCTN (ISRCTN38935454) The registration was completed after recruitment of the first patient, so the study was registered retrospectively.

Contributors: PJH wrote this manuscript; JN was involved in study design and protocol preparation; TJW was involved in study design and preparation of protocol amendments; NCB was involved in study design; ACS lead study design and protocol preparation. All authors reviewed this manuscript.

Funding: We are grateful to the Stoneygate Trust for partially funding this study. This work is supported by the National Institute for Health Research (NIHR) Diet, Lifestyle & Physical Activity Biomedical Research Unit based at University Hospitals of Leicester and Loughborough University. The views expressed are those of the authors and not necessarily those of the NHS, the NIHR or the Department of Health.

Competing interests: None declared.

Ethics Approval: REC East Midlands-Derby (ref 15/EM/0391).

References

1. Shlipak MG, Fried LF, Cushman M, Manolio T a, Peterson D, Stehman-Breen C, et al. Cardiovascular mortality risk in chronic kidney disease: comparison of traditional and novel risk factors. JAMA. 2005;293(14):1737–45.

2. Caskey F, Castledine C, Dawnay a, Farrington K, Fogarty D, Fraser S, et al. UK Renal Registry 2015 18th Annual Report of the Renal Association. Nephron [Internet]. 2016;132(suppl1):1–366. www.renalreg.org (accessed 27 Sep 2016).

3. Johansen KL, Shubert T, Doyle J, Soher B, Sakkas GK, Kent-Braun J a. Muscle atrophy in patients receiving hemodialysis: effects on muscle strength, muscle quality, and physical function. Kidney Int [Internet]. 2002;63(1):291–7.

For peer review only - http://bmjopen.bmj.com/site/about/guidelines.xhtml

http://www.nature.com/doifinder/10.1046/j.1523-1755.2003.00704.x\npapers3://publication/doi/10.1046/j.1523-1755.2003.00704.x (accessed 18 Jan 2016).

4. Fried LF, Lee JS, Shlipak M, Chertow GM, Green C, Ding J, et al. Chronic kidney disease and functional limitation in older people: health, aging and body composition study. J Am Geriatr Soc [Internet]. 2006;54(5):750–6. http://www.ncbi.nlm.nih.gov/pubmed/16696739 (accessed 15 Sep 2016).

5. Fouque D, Kalantar-Zadeh K, Kopple J, Cano N, Chauveau P, Cuppari L, et al. A proposed nomenclature and diagnostic criteria for protein-energy wasting in acute and chronic kidney disease. Kidney Int [Internet]. Elsevier Masson SAS; 2008;73(4):391–8. http://www.ncbi.nlm.nih.gov/pubmed/18094682 (15 Sep 2016).

6. Moreau K, Desseix A, Germain C, Barthe N, Bachelet T, Morel D, et al. Body composition in 98 patients awaiting kidney transplantation. Nutrition. 2014;30(2):186–91.

7. Johnson C, Gallagher-Lepak S, Zhu Y-R, Porth C, Kelber S, Roza A, et al. Factors influencing weight gain after renal transplantation. Transplantation. 1993;56(4):822–6.

8. Baum C. Weight Gain and Cardiovascular Risk After Organ Transplantation. J Parenter Enter Nutr [Internet]. 2001;25(3):114–9. http://www.thehastingscenter.org/Publications/BriefingBook/Detail.aspx?id=2198 (15 Sep 2016).

9. Van den Ham E, Kooman P, Leunissen KML, van Hooff JP. Posttransplantation weight gain is predominantly due to an increase in body fat mass. Transplantation. 2000;70(1):241–2.

10. Oberg BP, Mcmenamin E, Lucas FL, Mcmonagle E, Morrow J, Ikizler TA, et al. Increased prevalence of oxidant stress and inflammation in patients with moderate to severe chronic kidney disease. Kidney Int. 2004;65(3):1009–16.

11. Molnar MZ, Carrero JJ, Mucsi I, Remport A. Comparison of the malnutrition – inflammation score in chronic kidney disease patients and kidney transplant recipients. Int Urol Nephrol [Internet]. Springer Netherlands; 2015;1025–33. http://dx.doi.org/10.1007/s11255-015-0984-2 (accessed 8 Oct 2015).

12. Stenvinkel P, Heimbürger O, Paultre F, Diczfalusy U, Wang T, Berglund L, et al. Strong association between malnutrition, inflammation, and atherosclerosis in chronic renal failure. Kidney Int. 1999;55(5):1899–911.

13. Carrero JJ, Stenvinkel P, Cuppari L, Ikizler TA, Kalantar-Zadeh K, Kaysen G, et al. Etiology of the Protein-Energy Wasting Syndrome in Chronic Kidney Disease: A Consensus Statement From the International Society of Renal Nutrition and Metabolism (ISRNM). J Ren Nutr. 2013;23(2):77–90.

14. Hauser AB, Stinghen AEM, Kato S, Bucharles S, Aita C, Yuzawa Y, et al. Characteristics and causes of immune dysfunction related to uremia and dialysis. Perit Dial Int. 2008;28(SUPP. 3):183–7.

For peer review only

For peer review only - http://bmjopen.bmj.com/site/about/guidelines.xhtml

15. Girndt M, Sester U, Sester M, Kaul H, Köhler H. Impaired cellular immune function in patients with end-stage renal failure. Nephrol Dial Transplant. 1999;14(12):2807–10.

16. Hutchinson P, Chadban SJ, Atkins RC, Holdsworth SR. Laboratory assessment of immune function in renal transplant patients. Nephrol Dial Transplant [Internet]. 2003;18(5):983–9. http://ndt.oxfordjournals.org/cgi/doi/10.1093/ndt/gfg190 (accessed 28 Oct 2015).

17. Vereyken EJF, Kraaij MD, Baan CC, Rezaee F, Weimar W, Wood KJ, et al. A shift towards pro-inflammatory CD16+ monocyte subsets with preserved cytokine production potential after kidney transplantation. PLoS One [Internet]. 2013;8(7):e70152. http://journals.plos.org/plosone/article?id=10.1371/journal.pone.0070152 (1 Dec 2015).

18. Stehr MD, Von Lengerke T. Preventing weight gain through exercise and physical activity in the elderly: A systematic review. Maturitas [Internet]. Elsevier Ireland Ltd; 2012;72(1):13–22. http://dx.doi.org/10.1016/j.maturitas.2012.01.022 (15 Sep 2016).

19. Peterson MD, Sen A, Gordon PM. Influence of Resistance Exercise on Lean Body Mass in Aging Adults: A Meta-Analysis. Med Sci Sport Exerc. 2011;43(2):249–58.

20. De Vries NM, van Ravensberg CD, Hobbelen JSM, Olde Rikkert MGM, Staal JB, Nijhuis-van der Sanden MWG. Effects of physical exercise therapy on mobility, physical functioning, physical activity and quality of life in community-dwelling older adults with impaired mobility, physical disability and/or multi-morbidity: A meta-analysis. Ageing Res Rev [Internet]. Elsevier B.V.; 2012;11(1):136–49. http://dx.doi.org/10.1016/j.arr.2011.11.002 (accessed 15 Sep 2016).

21. Chou C-H, Hwang C-L, Wu Y-T. Effect of exercise on physical function, daily living activities, and quality of life in the frail older adults: a meta-analysis. Arch Phys Med Rehabil. 2012;93(2):237–44.

22. Woods J a., Vieira VJ, Keylock KT. Exercise, Inflammation, and Innate Immunity. Immunol Allergy Clin North Am. 2009;29(2):381–93.

23. Gleeson M, Bishop NC, Stensel DJ, Lindley MR, Mastana SS, Nimmo M a. The anti-inflammatory effects of exercise: mechanisms and implications for the prevention and treatment of disease. Nat Rev Immunol [Internet]. Nature Publishing Group; 2011;11(9):607–15. http://www.nature.com/doifinder/10.1038/nri3041 (accessed 6 Jan 2016).

24. Nieman DC, Pedersen BK. Exercise and Immune Function. Sport Med. 1999;27(2):73–80.

25. Walsh NP, Gleeson M, Shephard RJ, Gleeson M, Woods JA, Bishop NC, et al. Position statement part one : immune function and exercise. Exerc Immunol Rev. 2011;17(1):6–63.

26. Castaneda C, Layne JE, Munoz-Orians L, Gordon PL, Walsmith J, Foldvari M, et al. A Randomized Controlled Trial of Resistance Exercise Training to Improve Glycemic Control in Older Adults With Type 2 Diabetes. Diabetes Care. 2002;25(12):2335–41.

27. Mercer TH, Crawford C, Gleeson NP, Naish PF. Low-volume exercise rehabilitation improves functional capacity and self-reported functional status of dialysis patients. Am J Phys Med Rehabil [Internet]. 2002;81(3):162–7. http://www.ncbi.nlm.nih.gov/pubmed/11989511 (accessed 29 Apr 2015).

For peer review only

For peer review only - http://bmjopen.bmj.com/site/about/guidelines.xhtml

28. Koufaki P, Mercer TH, Naish PF. Effects of exercise training on aerobic and functional capacity of end-stage renal disease patients. Clin Physiol Funct Imaging. 2002;22(2):115–24.

29. Mustata S, Groeneveld S, Davidson W, Ford G, Kiland K, Manns B. Effects of exercise training on physical impairment , arterial stiffness and health-related quality of life in patients with chronic kidney disease : a pilot study. Int Urol Nephrol. 2011;43(1):1133–41.

30. Viana JL, Kosmadakis GC, Watson EL, Bevington a., Feehally J, Bishop NC, et al. Evidence for Anti-Inflammatory Effects of Exercise in CKD. J Am Soc Nephrol [Internet]. 2014;25(9):2121–30. http://www.jasn.org/cgi/doi/10.1681/ASN.2013070702 (accessed 12 Oct 2015).

31. Van Craenenbroeck AH, Van Ackeren K, Hoymans VY, Roeykens J, Verpooten G a., Vrints CJ, et al. Acute exercise-induced response of monocyte subtypes in chronic heart and renal failure. Mediators Inflamm. 2014;2014.

32. Singh SJ, Morgan MDL, Scott S, Walters D, Hardman AE. Development of a shuttle walking test of disability in patients with chronic airways obstruction. Thorax. 1992;47:1019–24.

33. Revill SM, Morgan MDL, Singh SJ, Williams J, Hardman a E. The endurance shuttle walk : a new field test for the assessment of endurance capacity in chronic obstructive pulmonary disease The endurance shuttle walk : a new field test for the assessment of endurance capacity in chronic obstructive pulmonary disease. Thorax. 1999;(July 2007):213–22.

34. Ziegler-Heitbrock L, Hofer TPJ. Toward a Refined Definition of Monocyte Subsets. Front Immunol [Internet]. 2013;4(February):1–5. http://journal.frontiersin.org/article/10.3389/fimmu.2013.00023/abstract (accessed 28 Jan 2015).

35. Eston R, Reilly T. Kinanthropometry and exercise physiology laboratory manual: anthropometry. 2009.

36. Chertow GM, Lowrie EG, Wilmore DW, Gonzalez J, Lew NL, Ling J, et al. Nutritional Assessment with Bioelectrical Impedance Analysis in Maintenance Hemodialysis Patients. J Am Soc Nephrol. 1995;6:75–81.

37. Squara P, Denjean D, Estagnasie P, Brusset A, Dib JC, Dubois C. Noninvasive cardiac output monitoring (NICOM): a clinical validation. Intensive Care Med. 2007;33:1191–4.

38. Obrador GT, Pereira BJG. Anaemia of chronic kidney disease : an under-recognized and under-treated problem. Nephrol Dial Transplant. 2002;177(S11):44–6.

39. Cheung WW, Paik KH, Mak RH. Inflammation and cachexia in chronic kidney disease. Pediatr Nephrol [Internet]. 2010;25(4):711–24. http://link.springer.com/10.1007/s00467-009-1427-z (accessed 1 Oct 2015).

40. Chao C-T, Huang J-W, Chiang C-K. Functional assessment of chronic illness therapy - the fatigue scale exhibits stronger associations with clinical parameters in chronic dialysis patients compared to other fatigue-assessing instruments. PeerJ. 2016;1818.

For peer review only

For peer review only - http://bmjopen.bmj.com/site/about/guidelines.xhtml

41. Jhamb M, Yabes J, Steel L, Amanda M, Unruh M. Prevalence and Correlates of Fatigue in Chronic Kidney Disease and End-Stage Renal Disease : Are Sleep Disorders a Key to Understanding Fatigue ? Am J Nephrol. 2013;38:489–95.

42. Gardner AW, Parker DE, Webb N, Montgomery PS, Scott KJ, Blevins SM, et al. Calf muscle hemoglobin oxygen saturation characteristics and exercise performance in patients with intermittent claudication. J Vasc Surg. 2008;48(3):644–9.

43. Wust RCI, Degens H. Factors contributing to muscle wasting and dysfunction in COPD patients. Int J COPD. 2007;2(3):289–300.

44. Grassi B, Quaresima V. Near-infrared spectroscopy and skeletal muscle oxidative function *in vivo* in health and disease: a review from an exercise physiology perspective. J Biomed Opt [Internet]. 2016;21(9):091313. http://biomedicaloptics.spiedigitallibrary.org/article.aspx?doi=10.1117/1.JBO.21.9.091313 (accessed 15 Sep 2016).

For peer review only

For peer review only - http://bmjopen.bmj.com/site/about/guidelines.xhtml

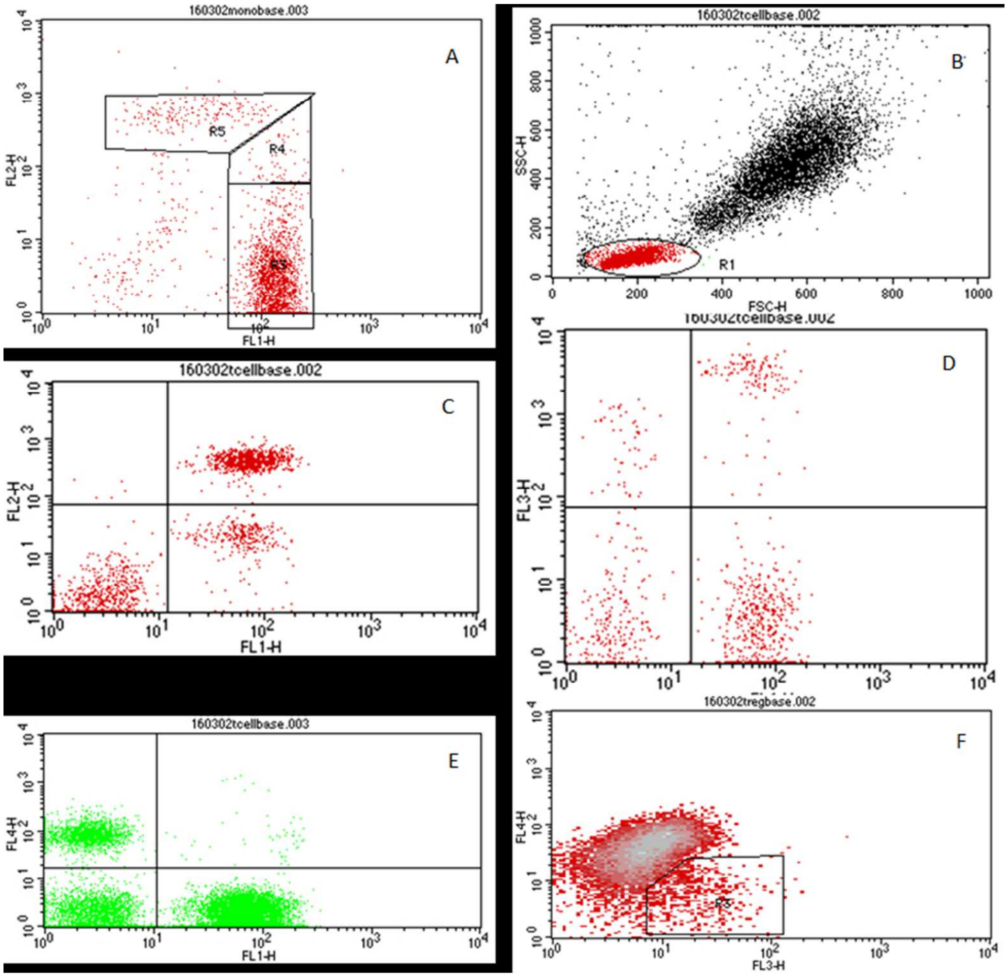

Figure 2. Demonstration of the gating strategy used to identify immune cell subsets. A: Monocyte subsets (R3 = classical (CD14++CD16-), R4 = intermediate (CD14++CD16+), R5 = non-classical (CD14+CD16++) on a CD14 (FL1) vs CD16 (FL2) plot. B: lymphocyte gating from standard forward vs. side scatter plot. C: Gating CD4+ helper T lymphocytes in the upper right quadrant, on a CD3 (FL1) vs CD4 (FL2) plot. D: Gating CD8+ cytotoxic T lymphocytes in the upper right quadrant, on a CD3 (FL1) vs. CD8 (FL3) plot. E: Gating B lymphocytes in the upper left quadrant, on a CD3 (FL1) vs. CD19 (FL4) plot. F: Gating regulatory T cells on a CD25 (FL3) vs. CD127 (FL4) plot. Not all graphs display 100% of acquired cells – this has been altered independently to allow ease of gating.

BMJ Open

Physical Activity, Immune Function and Inflammation in Kidney Patients : the PINK Study Protocol

Journal:	*BMJ Open*
Manuscript ID	bmjopen-2016-014713.R1
Article Type:	Protocol
Date Submitted by the Author:	12-Jan-2017
Complete List of Authors:	Highton, Patrick; Loughborough University, School of Sport, Exercise and Health Sciences; University of Leicester, Department of Infection, Immunity and Inflammation<br>Neale, Jill; University of Leicester, Department of Infection, Immunity and Inflammation<br>Wilkinson, Thomas; University of Leicester, Department of Infection, Immunity and Inflammation<br>Bishop , Nicolette ; Loughborough University, School of Sport, Exercise and Health Sciences; University of Leicester, Department of Infection, Immunity and Inflammation<br>Smith, Alice; University of Leicester, Department of Infection, Immunity and Inflammation; Loughborough University, School of Sport, Exercise and Health Sciences
**Primary Subject Heading**:	Immunology (including allergy)
Secondary Subject Heading:	Sports and exercise medicine, Renal medicine
Keywords:	Renal transplantation < NEPHROLOGY, IMMUNOLOGY, Inflammation, Exercise

SCHOLARONE™
Manuscripts

For peer review only

Physical Activity, Immune Function and Inflammation in Kidney Patients: the PINK Study Protocol

Patrick J Highton[1,2], Jill Neale[2], Thomas J Wilkinson[2], Nicolette C Bishop[1,2], Alice C Smith[1,2].

Corresponding author:

Patrick J Highton
Leicester Kidney Exercise Team
Academic Unit
Leicester General Hospital
LE5 4PW
P.Highton@lboro.ac.uk
0116 258 4346

[1] School of Sport, Exercise and Health Sciences, Loughborough University, Loughborough, United Kingdom

[2] Department of Infection, Immunity and Inflammation, University of Leicester, Leicester, United Kingdom

Keywords: renal transplantation, nephrology, immunology, inflammation, exercise

Word count (excluding title page, abstract, references, figures and tables): 1930

For peer review only - http://bmjopen.bmj.com/site/about/guidelines.xhtml

Abstract

Introduction: Chronic Kidney Disease (CKD) patients display increased infection-related mortality and elevated cardiovascular risk only partly attributed to traditional risk factors. CKD patients also exhibit a pro-inflammatory environment and impaired immune function. Aerobic exercise has the potential to positively impact these detriments, but is under-researched in this patient population. This feasibility study will investigate the effects of acute aerobic exercise on inflammation and immune function in CKD patients, to inform the design of larger studies intended to ultimately influence current exercise recommendations.

Methods and analysis: Patients with CKD, including renal transplant recipients, will visit the laboratory on two occasions, both preceded by appropriate exercise, alcohol and caffeine restrictions. On Visit 1, baseline assessments will be completed, comprising anthropometrics, body composition, cardiovascular function, and fatigue and leisure time exercise questionnaires. Participants will then undertake an incremental shuttle walk test, to estimate VO_2peak. On Visit 2, participants will complete a 20 minute shuttle walk at a constant speed to achieve 85% estimated VO_2peak. Blood and saliva samples will be taken before, immediately after and 1 hour after this exercise bout. Muscle O_2 saturation will be monitored throughout exercise and recovery. Age and sex-matched non-CKD 'healthy control' participants will complete an identical protocol. Blood and saliva samples will be analysed for markers of inflammation and immune function, using cytometric bead array and flow cytometry techniques. Appropriate statistical tests will be used to analyse the data.

Ethics and dissemination: A favourable opinion was granted by the East Midlands-Derby Research Ethics Committee on 18/09/2015 (ref 15/EM/0391) and the study was approved and sponsored by University Hospitals of Leicester Research and Innovation (ref 11444). The study was registered with ISRCTN (ref 38935454). The results will be presented at relevant conferences, and it is anticipated that the reports will be published in appropriate journals in 2018.

Strengths and limitations of this study
• Inclusion of control group matched for age and sex<br>• A variety of outcome measures to inform future study design<br>• Pragmatic study design which will inform future exercise recommendations for patients with CKD<br>• No non-exercise control visit

Introduction

Patients with all stages of Chronic Kidney Disease (CKD) have elevated cardiovascular disease (CVD) risk that cannot be fully explained by traditional risk factors (1). CVD is the most common cause of death amongst CKD patients (23% in 2013), followed by infection (19% in 2013) (2).

CKD patients are often sarcopenic and obese (3–6), and further deterioration in these characteristics is often observed after renal transplantation (7–9). Patients with CKD and renal transplant recipients (RTRs) also frequently suffer from chronic systemic inflammation (10,11), which can worsen cachexia and increase cardiovascular risk in CKD (12,13). CKD patients and RTRs also display impaired cellular immune function (14–16), which may explain why infection is the second-leading cause of death in this population (2). This effect (i.e. impaired immune function) is further compounded in RTRs by the immunosuppressive drug regime. Impaired immune function may

For peer review only - http://bmjopen.bmj.com/site/about/guidelines.xhtml

further exacerbate inflammation due to alterations in circulating immune cell subsets (17) which could serve to worsen body composition and increase cardiovascular risk. Finally, CKD patients display elevated levels of circulating pro-thrombotic microparticles (MPs) (18), which may worsen CVD risk and/or burden (19).

Exercise has the potential to benefit and improve many of the aforementioned inter-related morbidities. In the general population, exercise can positively impact upon weight gain (20), muscle wasting (21), physical capacity (22) and fatigue (23). Further, moderate aerobic exercise can also modify systemic inflammation (24,25) bolster immune function via alterations in circulating immune cell populations and activity, and reduce circulating MP levels (26–29).

In the CKD population, similar positive effects of exercise on body composition, physical function and quality of life have been demonstrated (30–33), however the research on inflammation and immune function in this population is limited. Previous research has shown that 30 minutes of moderate intensity walking exercise elicited a normal pattern of leukocyte and monocyte activation, whilst enhancing granulocyte function and promoting an anti-inflammatory environment (increased Interleukin-10 release) in pre-dialysis CKD patients (34). However, in a similar population an exhaustive bout of cycling exercise elicited a shift towards the more pro-inflammatory $CD16^+$ monocyte, potentially favouring inflammation (35). This disparity may be due to the exercise intensities investigated – moderate intensity exercise is more beneficial in strengthening immune function and preventing infection (26) and is promoted in general exercise guidelines. As such, investigating the effects of moderate intensity exercise in this patient population is more pragmatic and will help to guide future exercise recommendations, which are lacking in this patient population.

Therefore, this feasibility study aims investigate the effects of acute aerobic walking exercise on the inflammation and immune function in CKD patients. This will create the basis from which a larger trial can be conducted, including data for a power calculation, which will ultimately help to inform exercise recommendations in this population which are currently lacking, particularly with regards to immune function and inflammation (36). However, a preliminary power calculation (GPower 3.1) based upon the findings of Viana et al (34) suggests that 15 participants per group (e.g. pre-dialysis CKD, renal transplant recipients, healthy controls) will be sufficient.

Methods and Analysis

Outcomes to be measured
This study will investigate the effect of 20 minutes of moderate intensity walking exercise on immune cell subsets and inflammatory markers. Participants will be grouped based on their status (i.e. 'patient' or 'healthy control'). Both groups will complete an identical acute, cross-sectional study protocol necessitating two study visits, as explained below.

Participant recruitment
All CKD patients attending outpatient clinics within the University Hospitals of Leicester renal network will be screened for eligibility by their principle care provider prior to recruitment, and approached during their routine outpatient appointments. The inclusion and exclusion criteria for patients are summarised in Table 1. Healthy age and sex matched controls with no known chronic disease will be recruited from the local community. Those who do not believe themselves to suffer from any significant chronic disease will be eligible to participate. The 'broad' inclusion and exclusion criteria allows the incorporation of several different groups (i.e. non-dialysis CKD patients, dialysis patients, renal transplant recipients and healthy controls), and thus will allow comparison between

For peer review only - http://bmjopen.bmj.com/site/about/guidelines.xhtml

these groups. Similarly, the wide range of CKD stage will allow the influence of remaining renal function on the measured markers to be investigated.

Inclusion Criteria	Exclusion Criteria
▪ Established chronic kidney disease (all stages will be eligible including those with an established kidney transplant and those receiving dialysis treatment)	▪ Age under 18 years<br>▪ Pregnancy<br>▪ Received kidney transplant less than 6 months prior to study entry<br>▪ Any element of study assessment protocol considered by principle care provider to be contraindicated due to physical impairment, comorbidity or any other reason<br>▪ Inability to give informed consent for any reason<br>▪ Visual or hearing impairment or insufficient command of English to give informed consent or comply with the assessment protocol

Table 1. Inclusion and exclusion criteria for patients.

Trial design and timeline

This is a non-randomised, controlled, feasibility study. Participants will complete two study visits as described below.

Visit 1: Participants will arrive at the laboratory in the morning, unfasted but having consumed no caffeine or alcohol and completed no strenuous exercise for 24 hours. Participants will complete questionnaires about time spent in leisure activity and their perception of fatigue, and assessments of anthropometry, body composition and cardiovascular condition. Participants will then undertake the Incremental Shuttle Walk Test (ISWT), followed by the Endurance Shuttle Walk Test (ESWT) as explained below. If the participant cannot complete the full 20 minute duration of the ESWT, they will be withdrawn from the study.

Visit 2: Participants will arrive at the laboratory in the morning (8-10am, to minimise the influence of diurnal variation on immune parameters), following the same standardisation procedure as in Visit 1. After resting for 10 minutes, blood and saliva samples will be collected. The ESWT will then be completed at the same speed as in Visit 1, lasting for 20 minutes. Within 5 minutes of exercise cessation, another blood and saliva sample will be collected. The participant will then rest for 1 hour, after which a final blood and saliva sample will be collected. This protocol is summarised in a schematic in Figure 1, and the outcome measures are explained in greater detail below.

The time delay between recruitment, visit 1 and visit 2 will be kept to a minimum in order to prevent deconditioning and minimise dropout rates. For dialysis patients, both study visits will be completed on a non-dialysis day that is not after their 'long break' (i.e. weekend, or two consecutive days without dialysis based on their regular shift pattern), in order to minimise the effect of fluid overload.

Physical performance

Endurance Capacity will be assessed using the ISWT (37) and ESWT (38). In the ISWT, the participant walks a level 10m shuttle course at a speed controlled by an external audible bleep signal. The test

For peer review only - http://bmjopen.bmj.com/site/about/guidelines.xhtml

progressively increases at 1 minute intervals for a total of 12 intervals, and is terminated when the participant fails to complete a shuttle within the required time. Following the ISWT, a walking speed equating to 85% of predicted peak O_2 consumption (VO_2peak) can be calculated, using a conversion table based upon their ISWT performance. The ESWT is completed at this continuous speed on the same shuttle course until volitional exhaustion or the end of the test (20 minutes) is reached. For the purposes of this study, any participant who does not complete the full 20 minutes of the ESWT in Visit 1 will be excluded to ensure standardisation of the test in Visit 2. This protocol was initially developed for use in patients with chronic airways obstruction (37) but has been used in the CKD population (39,40), showing good reproducibility and a high correlation with VO_2peak (41).

Venous blood sampling

Venous blood will be collected using venepuncture of the antecubital vein of either arm – provided the absence of an arteriovenous fistula. Blood will be drawn through a 21-guage needle (30 ml per time-point) and collected into K_2EDTA (di-potassium ethylenediaminetetraacetic acid), sodium citrate, sodium heparin and blank monovettes.

Blood processing, storage and analysis

Blood collected into EDTA tubes will be centrifuged at 4ºC; the supernatant will then be aliquoted and frozen at -80ºC for future analysis. A cytometric bead array technique will be used to allow bulk-analysis of a panel of pro- and anti-inflammatory cytokines, including but not limited to IL-1, IL-2, IL-6, IL-10, TNF-α and IFNγ. Blood collected into sodium citrate (42) will be double-centrifuged at room temperature (15 mins at 2,500g, supernatant aliquoted followed by another 15 mins at 2,500g) to create platelet-poor plasma; the supernatant will then be aliquoted and frozen at -80ºC for future phenotyping of microparticles using flow cytometry, as explained below. Blood collected into sodium heparin will be analysed on the day of collection for immune cell subsets using flow cytometry, as explained below. Blood collected into blank monovettes will be sent to the Diagnostic Pathology Service at University Hospitals of Leicester NHS Trust on the day of collection for renal profile analysis, which includes eGFR, urea, bicarbonate, creatinine, sodium, potassium and phosphate measures. This will be completed for both patient and control populations.

Flow cytometry

Immune cells will be characterised based on their expression of surface antigens using flow cytometry. Monocyte subsets will be categorised as classical (CD14^{++}CD16^{-}), intermediate (CD14^{++}CD16^{+}) and non-classical (CD14^{+}CD16^{++}) (43). Monocytic ACE expression will be assessed using CD143, with IgG1 as a negative control. T cells will be categorised as helper (CD3^{+}CD4^{+}) cytotoxic (CD3^{+}CD8^{+}) and regulatory (CD4^{+}CD25^{+}CD127^{-}) T cells. B Cells will be identified as CD3^{+}CD19^{+} and NK Cells as CD3-CD56^{+}. Following appropriate staining and washing procedures, immune cells will be analysed on a FACS Calibur (BD Biosciences, Oxford, UK). Acquisition templates and prelimina[illegible]try results are displayed in Figure 2.

Microparticles will be characterised based on their size, Annexin-v expression, cellular derivation and pro-thrombotic potential. Cellular derivations will be categorised as platelet-derived (CD42b^{+}), neutrophil-derived (CD66b^{+}), monocyte-derived (CD14^{+}) and endothelial cell-derived (CD144^{+}). Pro-thrombotic potential will be estimated using Tissue Factor (CD142^{+}) expression. Following the thawing of platelet-free-plasma at room temperature, samples will be double-centrifuged at room temperature (30 mins at 18,000g, supernatant removed, discarded and replaced with an equal volume of buffer, followed by another 30 mins at 18,000g). Samples will then undergo appropriate staining procedures with the antibodies listed above, after which microparticles will be analysed on a BD Accuri C6 (BD Biosciences, Oxford, UK) flow cytometer. Acquisition templates and preliminary microparticle results are displayed in Figure 3. The protocol for isolating and analysing MPs is based upon a number of publications (42,44,45) and in our experience produces reliable results (Figure 3).

For peer review only

For peer review only - http://bmjopen.bmj.com/site/about/guidelines.xhtml

Saliva sampling and storage

Saliva samples will be collected into sterile plastic containers. Participants will swallow to empty the mouth, then open and hold the container themselves before performing a passive dribble of saliva collected under the tongue over the next two minutes. Following centrifugation, the supernatant will be aliquoted and frozen for future analysis, primarily for secretory Immunoglobulin A (sIgA) to investigate mucosal immunity.

Anthropometric measures

Height, weight and waist and hip circumference will be measured using standard procedures (46).

Body composition

Body composition parameters (i.e. fat and fat free mass content) will be measured using bioelectrical impedance analysis (BIA) (Inbody 370, Chicago, IL, USA). BIA has been validated for use in CKD (47).

Cardiovascular condition and function

Cardiac bioreactance analysis will be carried out using a Non-Invasive Cardiac Output Monitor (NICOM) (Cheetah Medical, Maidenhead, UK). Cardiac bioreactance is a quick, safe, validated (48), and non-invasive method of assessing central haemodynamics including heart rate, stroke volume, cardiac output, and total peripheral resistance. This is accomplished by using cutaneous electrodes placed on the chest in combination with an automatic sphygmomanometer to measure blood pressure.

Time spent in leisure activities

The Leisure Time Exercise Questionnaire (LTEQ) assesses the amount of exercise a patient undertakes, from which a metabolic equivalent (METS) estimation can be calculated. It also identifies preferred physical activities.

Fatigue

Fatigue is a common complaint CKD, and it has multiple potential mechanisms including anaemia (49) and cachexia (50). To assess self-reported fatigue, participants will be asked to fill in a fatigue scale (an internally designed Likert scale), as well as the validated Functional Assessment of Chronic Illness Therapy-Fatigue (FACIT-F) (51) which has been extensively used in CKD populations (52)

Muscle O_2 saturation

Reduced muscle O_2 saturation is another possible physiological mechanism of fatigue (53) and may be related to increased inflammation (54). A small non-invasive muscle O_2 saturation device (BSXInsight, BSXAthletics, TX, USA) will be fitted around the participant's calf and worn during exercise bouts and subsequent recovery. This device uses near infrared spectroscopy (NIRS) to measure haemoglobin and myoglobin oxygenation (muscle O_2 tissue stores). NIRS has previously been used in a variety of disease conditions (55).

Clinical information

Clinical records will be used to extract information that allows associations to be made with physical activity levels and account for confounding variables, including: age; gender; ethnicity; primary cause of renal failure; transplant or dialysis type; time since transplant or duration of dialysis; co-morbidities; and current medications.

Data analysis plan

Data analysis will compare groups at each time-point, investigate any changes elicited by exercise, and any other pertinent outcomes. Mixed-design ANOVAs will be used to analyse main effects of

For peer review only - http://bmjopen.bmj.com/site/about/guidelines.xhtml

group and time and interaction effects, with Bonferroni post-hoc testing used to elucidate these effects. This will generate both tests of significance and estimates of effect sizes. Any non-normally distributed data will first be transformed appropriately to ensure normality assumptions are met, to increase statistical power and to allow clear conclusions to be drawn from the data. This will allow the generation of hypotheses for future, larger studies.

Ethics and dissemination

Ethical and safety considerations

This protocol was reviewed by the East Midlands-Derby Research Ethics Committee (REC) and was given a favourable opinion (REC ref 15/EM/0391) on 24/09/2015. Additionally, local approval was given by the Research and Innovation office at the University Hospitals of Leicester NHS Trust (ref 11444) on 29/09/2015. Therefore, all steps have been taken when designing this protocol to minimise all ethical implications and ensure patient welfare. Two Substantial Amendments were made to this protocol, approved on 03/12/15 and 01/08/16 to add saliva collection, and fatigue questionnaires and muscle O_2 saturation respectively. The protocol presented here represents the most up to date version.

Dissemination plan

The results are planned for publication in early 2018. We plan to present the data at relevant national and international conferences, as well as publish the findings in relevant journals. Participant level data will be available at a later date.

Trial registration

This study is registered with the ISRCTN (ISRCTN38935454) The registration was completed after recruitment of the first patient, so the study was registered retrospectively.

Contributors: PJH wrote this manuscript; JN was involved in study design and protocol preparation; TJW was involved in study design and preparation of protocol amendments; NCB was involved in study design; ACS lead study design and protocol preparation. All authors reviewed this manuscript.

Funding: We are grateful to the Stoneygate Trust for partially funding this study. This work is supported by the National Institute for Health Research (NIHR) Diet, Lifestyle & Physical Activity Biomedical Research Unit based at University Hospitals of Leicester and Loughborough University. The views expressed are those of the authors and not necessarily those of the NHS, the NIHR or the Department of Health.

Competing interests: None declared.

Ethics Approval: REC East Midlands-Derby (ref 15/EM/0391).

References

1. Shlipak MG, Fried LF, Cushman M, Manolio T a, Peterson D, Stehman-Breen C, et al. Cardiovascular mortality risk in chronic kidney disease: comparison of traditional and novel risk factors. JAMA. 2005;293(14):1737–45.

For peer review only - http://bmjopen.bmj.com/site/about/guidelines.xhtml

2. Caskey F, Castledine C, Dawnay a, Farrington K, Fogarty D, Fraser S, et al. UK Renal Registry 2015 18th Annual Report of the Renal Association. Nephron [Internet]. 2016;132(suppl1):1–366. Available from: www.renalreg.org

3. Johansen KL, Shubert T, Doyle J, Soher B, Sakkas GK, Kent-Braun J a. Muscle atrophy in patients receiving hemodialysis: effects on muscle strength, muscle quality, and physical function. Kidney Int [Internet]. 2002;63(1):291–7. Available from: http://www.nature.com/doifinder/10.1046/j.1523-1755.2003.00704.x\npapers3://publication/doi/10.1046/j.1523-1755.2003.00704.x

4. Fried LF, Lee JS, Shlipak M, Chertow GM, Green C, Ding J, et al. Chronic kidney disease and functional limitation in older people: health, aging and body composition study. J Am Geriatr Soc [Internet]. 2006;54(5):750–6. Available from: http://www.ncbi.nlm.nih.gov/pubmed/16696739

5. Fouque D, Kalantar-Zadeh K, Kopple J, Cano N, Chauveau P, Cuppari L, et al. A proposed nomenclature and diagnostic criteria for protein-energy wasting in acute and chronic kidney disease. Kidney Int [Internet]. Elsevier Masson SAS; 2008;73(4):391–8. Available from: http://www.ncbi.nlm.nih.gov/pubmed/18094682

6. Moreau K, Desseix A, Germain C, Barthe N, Bachelet T, Morel D, et al. Body composition in 98 patients awaiting kidney transplantation. Nutrition. 2014;30(2):186–91.

7. Johnson C, Gallagher-Lepak S, Zhu Y-R, Porth C, Kelber S, Roza A, et al. Factors influencing weight gain after renal transplantation. Transplantation. 1993;56(4):822–6.

8. Baum C. Weight Gain and Cardiovascular Risk After Organ Transplantation. J Parenter Enter Nutr [Internet]. 2001;25(3):114–9. Available from: http://www.thehastingscenter.org/Publications/BriefingBook/Detail.aspx?id=2198

9. van den Ham E, Kooman P, Leunissen KML, van Hooff JP. Posttransplantation weight gain is predominantly due to an increase in body fat mass. Transplantation. 2000;70(1):241–2.

10. Oberg BP, Mcmenamin E, Lucas FL, Mcmonagle E, Morrow J, Ikizler TA, et al. Increased prevalence of oxidant stress and inflammation in patients with moderate to severe chronic kidney disease. Kidney Int. 2004;65(3):1009–16.

11. Molnar MZ, Carrero JJ, Mucsi I, Remport A. Comparison of the malnutrition – inflammation score in chronic kidney disease patients and kidney transplant recipients. Int Urol Nephrol [Internet]. Springer Netherlands; 2015;1025–33. Available from: "http://dx.doi.org/10.1007/s11255-015-0984-2

12. Stenvinkel P, Heimbürger O, Paultre F, Diczfalusy U, Wang T, Berglund L, et al. Strong association between malnutrition, inflammation, and atherosclerosis in chronic renal failure. Kidney Int. 1999;55(5):1899–911.

13. Carrero JJ, Stenvinkel P, Cuppari L, Ikizler TA, Kalantar-Zadeh K, Kaysen G, et al. Etiology of the Protein-Energy Wasting Syndrome in Chronic Kidney Disease: A Consensus Statement From the International Society of Renal Nutrition and Metabolism (ISRNM). J Ren Nutr. 2013;23(2):77–90.

14. Hauser AB, Stinghen AEM, Kato S, Bucharles S, Aita C, Yuzawa Y, et al. Characteristics and causes of immune dysfunction related to uremia and dialysis. Perit Dial Int. 2008;28(SUPP. 3):183–7.

15. Girndt M, Sester U, Sester M, Kaul H, Köhler H. Impaired cellular immune function in patients with end-stage renal failure. Nephrol Dial Transplant. 1999;14(12):2807–10.

For peer review only - http://bmjopen.bmj.com/site/about/guidelines.xhtml

16. Hutchinson P, Chadban SJ, Atkins RC, Holdsworth SR. Laboratory assessment of immune function in renal transplant patients. Nephrol Dial Transplant [Internet]. 2003;18(5):983–9. Available from: http://ndt.oxfordjournals.org/cgi/doi/10.1093/ndt/gfg190

17. Vereyken EJF, Kraaij MD, Baan CC, Rezaee F, Weimar W, Wood KJ, et al. A shift towards pro-inflammatory CD16+ monocyte subsets with preserved cytokine production potential after kidney transplantation. PLoS One [Internet]. 2013;8(7):e70152. Available from: http://journals.plos.org/plosone/article?id=10.1371/journal.pone.0070152

18. Lu GY, Xu RJ, Zhang SH, Qiao Q, Shen L, Li M, et al. Alteration of circulatory platelet microparticles and endothelial microparticles in patients with chronic kidney disease. Int J Clin Exp Med. 2015;8(9):16704–8.

19. Vanwijk MJ, Vanbavel E, Sturk a, Nieuwland R. M icroparticles in cardiovascular diseases. Cardiovasc Res. 2003;59:277–87.

20. Stehr MD, Von Lengerke T. Preventing weight gain through exercise and physical activity in the elderly: A systematic review. Maturitas [Internet]. Elsevier Ireland Ltd; 2012;72(1):13–22. Available from: http://dx.doi.org/10.1016/j.maturitas.2012.01.022

21. Peterson MD, Sen A, Gordon PM. Influence of Resistance Exercise on Lean Body Mass in Aging Adults: A Meta-Analysis. Med Sci Sport Exerc. 2011;43(2):249–58.

22. de Vries NM, van Ravensberg CD, Hobbelen JSM, Olde Rikkert MGM, Staal JB, Nijhuis-van der Sanden MWG. Effects of physical exercise therapy on mobility, physical functioning, physical activity and quality of life in community-dwelling older adults with impaired mobility, physical disability and/or multi-morbidity: A meta-analysis. Ageing Res Rev [Internet]. Elsevier B.V.; 2012;11(1):136–49. Available from: http://dx.doi.org/10.1016/j.arr.2011.11.002

23. Chou C-H, Hwang C-L, Wu Y-T. Effect of Exercise on Physical Function, Daily Living Activities, and Quality of Life in the Frail Older Adults: A Meta-Analysis. Arch Phys Med Rehabil. 2012;93(2):237–44.

24. Woods J a., Vieira VJ, Keylock KT. Exercise, Inflammation, and Innate Immunity. Immunol Allergy Clin North Am. 2009;29(2):381–93.

25. Gleeson M, Bishop NC, Stensel DJ, Lindley MR, Mastana SS, Nimmo M a. The anti-inflammatory effects of exercise: mechanisms and implications for the prevention and treatment of disease. Nat Rev Immunol [Internet]. Nature Publishing Group; 2011;11(9):607–15. Available from: http://www.nature.com/doifinder/10.1038/nri3041

26. Nieman DC, Pedersen BK. Exercise and Immune Function. Sport Med. 1999;27(2):73–80.

27. Walsh NP, Gleeson M, Shephard RJ, Gleeson M, Woods JA, Bishop NC, et al. Position statement part one : immune function and exercise. Exerc Immunol Rev. 2011;17(1):6–63.

28. Babbitt DM, Diaz KM, Feairheller DL, Sturgeon KM, Perkins AM, Veerabhadrappa P, et al. Endothelial activation microparticles and inflammation status improve with exercise training in african americans. Int J Hypertens [Internet]. 2013;2013:538017. Available from: http://www.pubmedcentral.nih.gov/articlerender.fcgi?artid=3652180&tool=pmcentrez&rendertype=abstract

29. Wahl P, Jansen F, Achtzehn S, Schmitz T, Bloch W, Mester J, et al. Effects of High Intensity Training and High Volume Training on Endothelial Microparticles and Angiogenic Growth Factors. PLoS One [Internet]. 2014;9(4):e96024. Available from: http://dx.plos.org/10.1371/journal.pone.0096024

For peer review only

30. Castaneda C, Layne JE, Munoz-Orians L, Gordon PL, Walsmith J, Foldvari M, et al. A Randomized Controlled Trial of Resistance Exercise Training to Improve Glycemic Control in Older Adults With Type 2 Diabetes. Diabetes Care. 2002;25(12):2335–41.

31. Mercer TH, Crawford C, Gleeson NP, Naish PF. Low-volume exercise rehabilitation improves functional capacity and self-reported functional status of dialysis patients. Am J Phys Med Rehabil [Internet]. 2002;81(3):162–7. Available from: http://www.ncbi.nlm.nih.gov/pubmed/11989511

32. Koufaki P, Mercer TH, Naish PF. Effects of exercise training on aerobic and functional capacity of end-stage renal disease patients. Clin Physiol Funct Imaging. 2002;22(2):115–24.

33. Mustata S, Groeneveld S, Davidson W, Ford G, Kiland K, Manns B. Effects of exercise training on physical impairment , arterial stiffness and health-related quality of life in patients with chronic kidney disease : a pilot study. Int Urol Nephrol. 2011;43(1):1133–41.

34. Viana JL, Kosmadakis GC, Watson EL, Bevington a., Feehally J, Bishop NC, et al. Evidence for Anti-Inflammatory Effects of Exercise in CKD. J Am Soc Nephrol [Internet]. 2014;25(9):2121–30. Available from: http://www.jasn.org/cgi/doi/10.1681/ASN.2013070702

35. Van Craenenbroeck AH, Van Ackeren K, Hoymans VY, Roeykens J, Verpooten G a., Vrints CJ, et al. Acute exercise-induced response of monocyte subtypes in chronic heart and renal failure. Mediators Inflamm. 2014;2014.

36. Johansen KL. Exercise and chronic kidney disease: current recommendations. Sport Med. 2005;35(6):485–99.

37. Singh SJ, Morgan MDL, Scott S, Walters D, Hardman AE. Development of a shuttle walking test of disability in patients with chronic airways obstruction. Thorax. 1992;47:1019–24.

38. Revill SM, Morgan MDL, Singh SJ, Williams J, Hardman a E. The endurance shuttle walk : a new field test for the assessment of endurance capacity in chronic obstructive pulmonary disease The endurance shuttle walk : a new field test for the assessment of endurance capacity in chronic obstructive pulmonary disease. Thorax. 1999;(July 2007):213–22.

39. Greenwood S a., Lindup H, Taylor K, Koufaki P, Rush R, MacDougall IC, et al. Evaluation of a pragmatic exercise rehabilitation programme in chronic kidney disease. Nephrol Dial Transplant. 2012;27(SUPPL. 3).

40. Wilund KR, Tomayko EJ, Wu P-T, Ryong Chung H, Vallurupalli S, Lakshminarayanan B, et al. Intradialytic exercise training reduces oxidative stress and epicardial fat: a pilot study. Nephrol Dial Transplant [Internet]. 2010;25(8):2695–701. Available from: http://ndt.oxfordjournals.org/cgi/doi/10.1093/ndt/gfq106

41. Painter P, Marcus RL. Assessing physical function and physical activity in patients with CKD. Clin J Am Soc Nephrol [Internet]. 2013;8(5):861–72. Available from: http://www.ncbi.nlm.nih.gov/pubmed/23220421

42. van der Heyde HC, Gramaglia I, Combes V, George TC, Grau GE. Flow Cytometric Analysis of Microparticles. Methods Mol Biol. 2011;699:337–54.

43. Ziegler-Heitbrock L, Hofer TPJ. Toward a Refined Definition of Monocyte Subsets. Front Immunol [Internet]. 2013;4(February):1–5. Available from: http://journal.frontiersin.org/article/10.3389/fimmu.2013.00023/abstract

44. Nielsen MH, Beck-Nielsen H, Andersen MN, Handberg A. A flow cytometric method for characterization of circulating cell-derived microparticles in plasma. J Extracell vesicles

For peer review only - http://bmjopen.bmj.com/site/about/guidelines.xhtml

[Internet]. 2014;3:1–12. Available from: http://www.pubmedcentral.nih.gov/articlerender.fcgi?artid=3916676&tool=pmcentrez&rendertype=abstract

45. Jy W, Horstman L, Jiminez J, Ahn YS. Measuring circulating cell-derived microparticles. J Thromb Haemost. 2004;2(1):1842–51.

46. Eston R, Reilly T. Kinanthropometry and exercise physiology laboratory manual: anthropometry. 2009.

47. Chertow GM, Lowrie EG, Wilmore DW, Gonzalez J, Lew NL, Ling J, et al. Nutritional Assessment with Bioelectrical Impedance Analysis in Maintenance Hemodialysis Patients. J Am Soc Nephrol. 1995;6:75–81.

48. Squara P, Denjean D, Estagnasie P, Brusset A, Dib JC, Dubois C. Noninvasive cardiac output monitoring (NICOM): a clinical validation. Intensive Care Med. 2007;33:1191–4.

49. Obrador GT, Pereira BJG. Anaemia of chronic kidney disease : an under-recognized and under-treated problem. Nephrol Dial Transplant. 2002;177(S11):44–6.

50. Cheung WW, Paik KH, Mak RH. Inflammation and cachexia in chronic kidney disease. Pediatr Nephrol [Internet]. 2010;25(4):711–24. Available from: http://link.springer.com/10.1007/s00467-009-1427-z

51. Chao C-T, Huang J-W, Chiang C-K. Functional assessment of chronic illness therapy - the fatigue scale exhibits stronger associations with clinical parameters in chronic dialysis patients compared to other fatigue-assessing instruments. PeerJ. 2016;1818.

52. Jhamb M, Yabes J, Steel L, Amanda M, Unruh M. Prevalence and Correlates of Fatigue in Chronic Kidney Disease and End-Stage Renal Disease : Are Sleep Disorders a Key to Understanding Fatigue ? Am J Nephrol. 2013;38:489–95.

53. Gardner AW, Parker DE, Webb N, Montgomery PS, Scott KJ, Blevins SM, et al. Calf muscle hemoglobin oxygen saturation characteristics and exercise performance in patients with intermittent claudication. J Vasc Surg. 2008;48(3):644–9.

54. Wust RCI, Degens H. Factors contributing to muscle wasting and dysfunction in COPD patients. Int J COPD. 2007;2(3):289–300.

55. Grassi B, Quaresima V. Near-infrared spectroscopy and skeletal muscle oxidative function *in vivo* in health and disease: a review from an exercise physiology perspective. J Biomed Opt [Internet]. 2016;21(9):91313. Available from: http://biomedicaloptics.spiedigitallibrary.org/article.aspx?doi=10.1117/1.JBO.21.9.091313

For peer review only

For peer review only - http://bmjopen.bmj.com/site/about/guidelines.xhtml

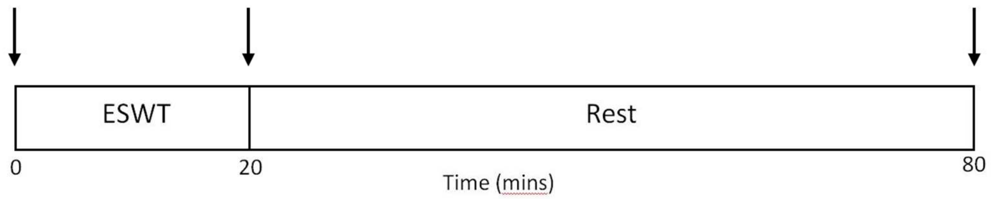

Figure 1. Visit 2 exercise and sample collection protocol. ↓= Venous blood and saliva collection. ESWT = Endurance Shuttle Walk Test

111x23mm (300 x 300 DPI)

For peer review only

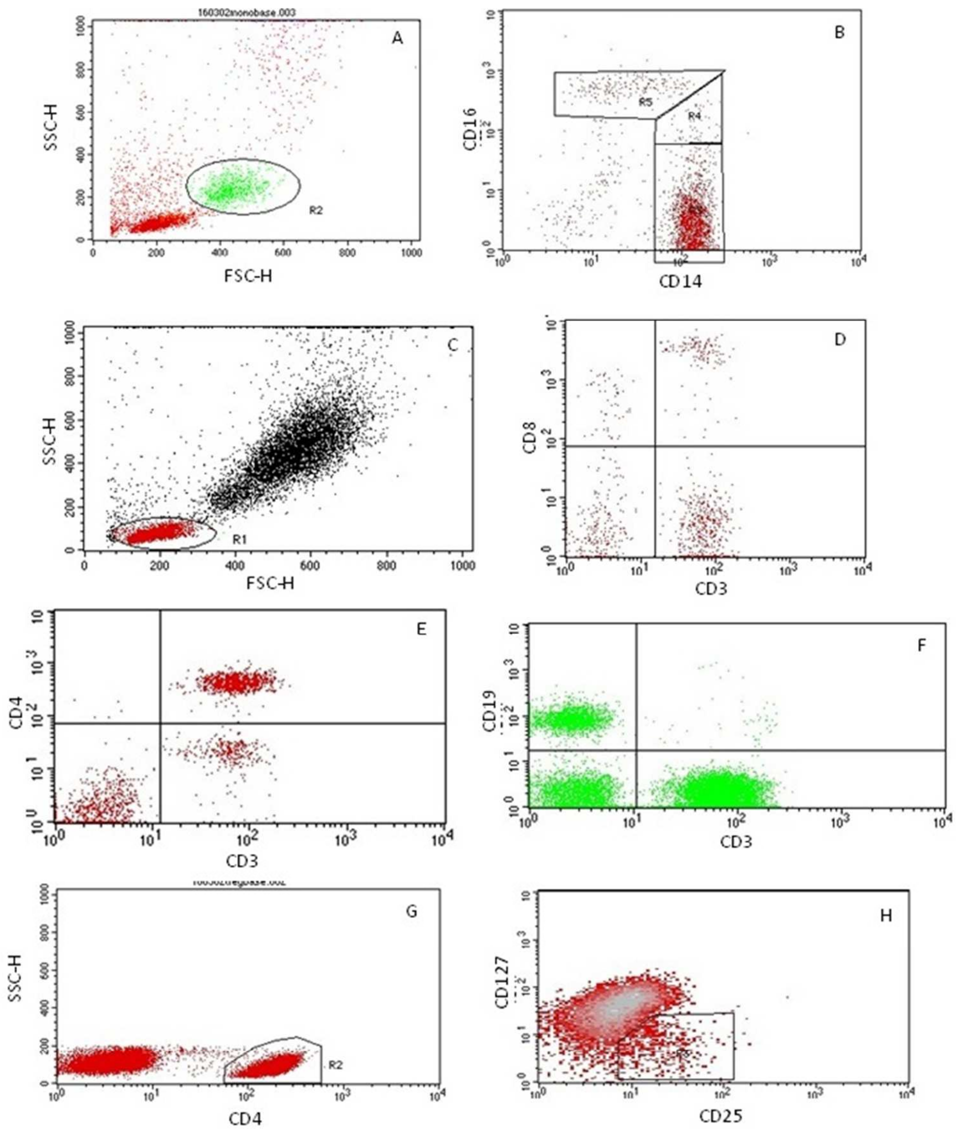

Figure 2. Demonstration of the gating strategy used to identify immune cell subsets. A: Total monocyte gating. B: Monocyte subsets (R3 = classical (CD14++CD16-), R4 = intermediate (CD14++CD16+), R5 = non-classical (CD14+CD16++). C: Total lymphocyte gating. D: Gating CD8+ cytotoxic T lymphocytes in the upper right quadrant. E: Gating CD4+ helper T lymphocytes in the upper right quadrant. F: Gating B lymphocytes (CD3-CD19+) in the upper left quadrant. G:Initial T-Reg gating, identifying CD4+ lymphocytes. H: Secondary T-reg gating, back-gated onto plot G, further identifying the CD4+ lymphocytes that are CD25+CD127-. Not all graphs display 100% of acquired cells – this has been altered independently to allow ease of gating.

61x70mm (300 x 300 DPI)

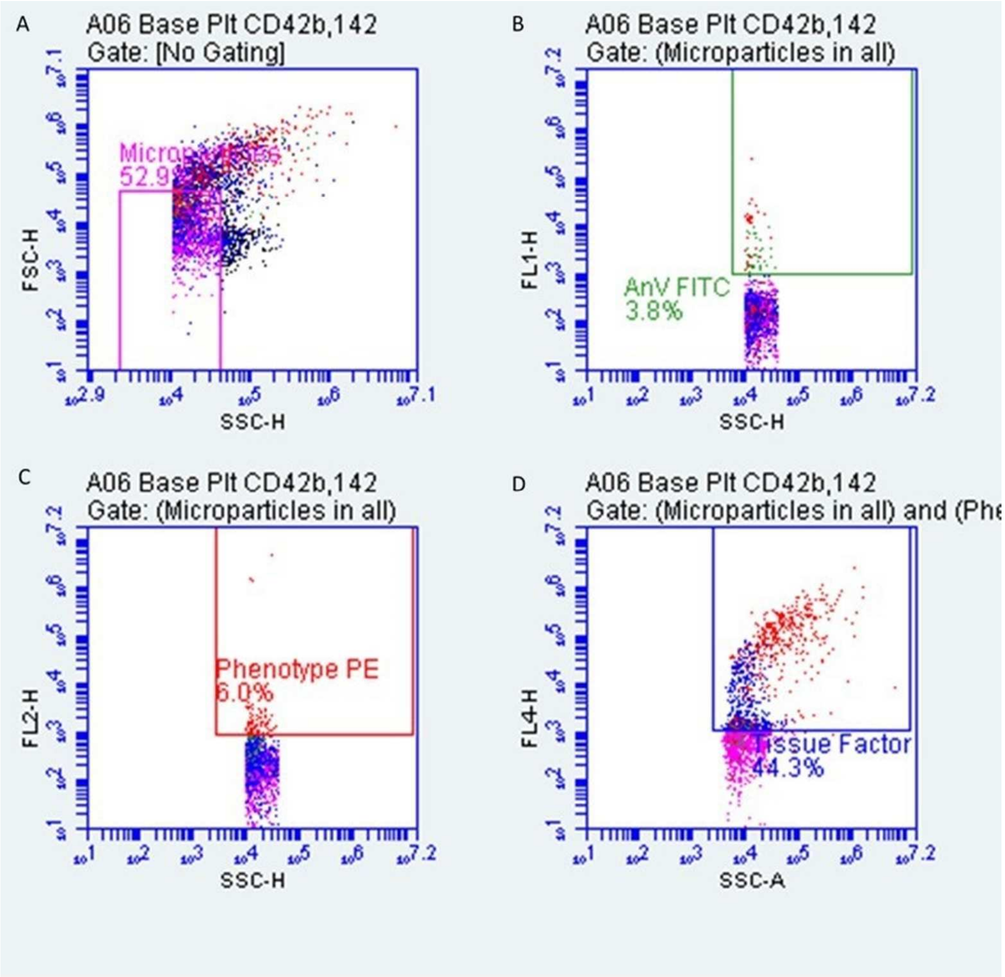

Figure 3. Demonstration of the gating strategy used to characterise microparticles. A: ‘All microparticles’, set using beads of known size. B: ‘All microparticles’ based on Annexin-v expression. C: Phenotype marker expression, used to quantify microparticles of different cellular sources. D: Pro-thrombotic potential, assessed by Tissue Factor expression. All gates have been appropriately sized and positioned using unstained samples to distinguish negative vs positive staining.

66x64mm (300 x 300 DPI)

For peer review only - http://bmjopen.bmj.com/site/about/guidelines.xhtml

BMJ Open

Physical Activity, Immune Function and Inflammation in Kidney Patients : the PINK Study Protocol

Journal:	*BMJ Open*
Manuscript ID	bmjopen-2016-014713.R2
Article Type:	Protocol
Date Submitted by the Author:	25-Jan-2017
Complete List of Authors:	Highton, Patrick; Loughborough University, School of Sport, Exercise and Health Sciences; University of Leicester, Department of Infection, Immunity and Inflammation<br>Neale, Jill; University of Leicester, Department of Infection, Immunity and Inflammation<br>Wilkinson, Thomas; University of Leicester, Department of Infection, Immunity and Inflammation<br>Bishop , Nicolette ; Loughborough University, School of Sport, Exercise and Health Sciences; University of Leicester, Department of Infection, Immunity and Inflammation<br>Smith, Alice; University of Leicester, Department of Infection, Immunity and Inflammation; Loughborough University, School of Sport, Exercise and Health Sciences
**Primary Subject Heading**:	Immunology (including allergy)
Secondary Subject Heading:	Sports and exercise medicine, Renal medicine
Keywords:	Renal transplantation < NEPHROLOGY, IMMUNOLOGY, Inflammation, Exercise

SCHOLARONE™
Manuscripts

Physical Activity, Immune Function and Inflammation in Kidney Patients: the PINK Study Protocol

Patrick J Highton[1,2], Jill Neale[2], Thomas J Wilkinson[2], Nicolette C Bishop[1,2], Alice C Smith[1,2].

Corresponding author:

Patrick J Highton
Leicester Kidney Exercise Team
Academic Unit
Leicester General Hospital
LE5 4PW
P.Highton@lboro.ac.uk
0116 258 4346

[1] School of Sport, Exercise and Health Sciences, Loughborough University, Loughborough, United Kingdom

[2] Department of Infection, Immunity and Inflammation, University of Leicester, Leicester, United Kingdom

Keywords: renal transplantation, nephrology, immunology, inflammation, exercise

Word count (excluding title page, abstract, references, figures and tables): 1930

For peer review only

Abstract

Introduction: Chronic Kidney Disease (CKD) patients display increased infection-related mortality and elevated cardiovascular risk only partly attributed to traditional risk factors. CKD patients also exhibit a pro-inflammatory environment and impaired immune function. Aerobic exercise has the potential to positively impact these detriments, but is under-researched in this patient population. This feasibility study will investigate the effects of acute aerobic exercise on inflammation and immune function in CKD patients, to inform the design of larger studies intended to ultimately influence current exercise recommendations.

Methods and analysis: Patients with CKD, including renal transplant recipients, will visit the laboratory on two occasions, both preceded by appropriate exercise, alcohol and caffeine restrictions. On Visit 1, baseline assessments will be completed, comprising anthropometrics, body composition, cardiovascular function, and fatigue and leisure time exercise questionnaires. Participants will then undertake an incremental shuttle walk test, to estimate VO_2peak. On Visit 2, participants will complete a 20 minute shuttle walk at a constant speed to achieve 85% estimated VO_2peak. Blood and saliva samples will be taken before, immediately after and 1 hour after this exercise bout. Muscle O_2 saturation will be monitored throughout exercise and recovery. Age and sex-matched non-CKD 'healthy control' participants will complete an identical protocol. Blood and saliva samples will be analysed for markers of inflammation and immune function, using cytometric bead array and flow cytometry techniques. Appropriate statistical tests will be used to analyse the data.

Ethics and dissemination: A favourable opinion was granted by the East Midlands-Derby Research Ethics Committee on 18/09/2015 (ref 15/EM/0391) and the study was approved and sponsored by University Hospitals of Leicester Research and Innovation (ref 11444). The study was registered with ISRCTN (ref 38935454). The results will be presented at relevant conferences, and it is anticipated that the reports will be published in appropriate journals in 2018.

Strengths and limitations of this study
• Inclusion of control group matched for age and sex<br>• A variety of outcome measures to inform future study design<br>• Pragmatic study design which will inform future exercise recommendations for patients with CKD<br>• No non-exercise control visit

Introduction

Patients with all stages of Chronic Kidney Disease (CKD) have elevated cardiovascular disease (CVD) risk that cannot be fully explained by traditional risk factors (1). CVD is the most common cause of death amongst CKD patients (23% in 2013), followed by infection (19% in 2013) (2).

CKD patients are often sarcopenic and obese (3–6), and further deterioration in these characteristics is often observed after renal transplantation (7–9). Patients with CKD and renal transplant recipients (RTRs) also frequently suffer from chronic systemic inflammation (10,11), which can worsen cachexia and increase cardiovascular risk in CKD (12,13). CKD patients and RTRs also display impaired cellular immune function (14–16), which may explain why infection is the second-leading cause of death in this population (2). This effect (i.e. impaired immune function) is further compounded in RTRs by the immunosuppressive drug regime. Impaired immune function may

For peer review only - http://bmjopen.bmj.com/site/about/guidelines.xhtml

further exacerbate inflammation due to alterations in circulating immune cell subsets (17) which could serve to worsen body composition and increase cardiovascular risk. Finally, CKD patients display elevated levels of circulating pro-thrombotic microparticles (MPs) (18), which may worsen CVD risk and/or burden (19).

Exercise has the potential to benefit and improve many of the aforementioned inter-related morbidities. In the general population, exercise can positively impact upon weight gain (20), muscle wasting (21), physical capacity (22) and fatigue (23). Further, moderate aerobic exercise can also modify systemic inflammation (24,25) bolster immune function via alterations in circulating immune cell populations and activity, and reduce circulating MP levels (26–29).

In the CKD population, similar positive effects of exercise on body composition, physical function and quality of life have been demonstrated (30–33), however the research on inflammation and immune function in this population is limited. Previous research has shown that 30 minutes of moderate intensity walking exercise elicited a normal pattern of leukocyte and monocyte activation, whilst enhancing granulocyte function and promoting an anti-inflammatory environment (increased Interleukin-10 release) in pre-dialysis CKD patients (34). However, in a similar population an exhaustive bout of cycling exercise elicited a shift towards the more pro-inflammatory $CD16^+$ monocyte, potentially favouring inflammation (35). This disparity may be due to the exercise intensities investigated – moderate intensity exercise is more beneficial in strengthening immune function and preventing infection (26) and is promoted in general exercise guidelines. As such, investigating the effects of moderate intensity exercise in this patient population is more pragmatic and will help to guide future exercise recommendations, which are lacking in this patient population.

Therefore, this feasibility study aims investigate the effects of acute aerobic walking exercise on the inflammation and immune function in CKD patients. This will create the basis from which a larger trial can be conducted, including data for a power calculation, which will ultimately help to inform exercise recommendations in this population which are currently lacking, particularly with regards to immune function and inflammation (36). However, a preliminary power calculation (GPower 3.1) based upon the findings of Viana et al (34) concerning total lymphocyte concentration (based on a statistical power of 0.80 and an alpha level of 0.05) suggests that 15 participants per group (e.g. pre-dialysis CKD, renal transplant recipients, healthy controls) will be sufficient.

Methods and Analysis

Outcomes to be measured

This study will investigate the effect of 20 minutes of moderate intensity walking exercise on immune cell subsets and inflammatory markers. Participants will be grouped based on their status (i.e. 'patient' or 'healthy control'). Both groups will complete an identical acute, cross-sectional study protocol necessitating two study visits, as explained below.

Participant recruitment

All CKD patients attending outpatient clinics within the University Hospitals of Leicester renal network will be screened for eligibility by their principle care provider prior to recruitment, and approached during their routine outpatient appointments. The inclusion and exclusion criteria for patients are summarised in Table 1. Healthy age and sex matched controls with no known chronic disease will be recruited from the local community. Those who do not believe themselves to suffer from any significant chronic disease will be eligible to participate. The 'broad' inclusion and exclusion criteria allows the incorporation of several different groups (i.e. non-dialysis CKD patients, dialysis

For peer review only - http://bmjopen.bmj.com/site/about/guidelines.xhtml

patients, renal transplant recipients and healthy controls), and thus will allow comparison between these groups. Similarly, the wide range of CKD stage will allow the influence of remaining renal function on the measured markers to be investigated.

Inclusion Criteria	Exclusion Criteria
▪ Established chronic kidney disease (all stages will be eligible including those with an established kidney transplant and those receiving dialysis treatment)	▪ Age under 18 years<br>▪ Pregnancy<br>▪ Received kidney transplant less than 6 months prior to study entry<br>▪ Any element of study assessment protocol considered by principle care provider to be contraindicated due to physical impairment, comorbidity or any other reason<br>▪ Inability to give informed consent for any reason<br>▪ Visual or hearing impairment or insufficient command of English to give informed consent or comply with the assessment protocol

Table 1. Inclusion and exclusion criteria for patients.

Trial design and timeline

This is a non-randomised, controlled, feasibility study. Participants will complete two study visits as described below.

Visit 1: Participants will arrive at the laboratory in the morning, unfasted but having consumed no caffeine or alcohol and completed no strenuous exercise for 24 hours. Participants will complete questionnaires about time spent in leisure activity and their perception of fatigue, and assessments of anthropometry, body composition and cardiovascular condition. Participants will then undertake the Incremental Shuttle Walk Test (ISWT), followed by the Endurance Shuttle Walk Test (ESWT) as explained below. If the participant cannot complete the full 20 minute duration of the ESWT, they will be withdrawn from the study.

Visit 2: Participants will arrive at the laboratory in the morning (8-10am, to minimise the influence of diurnal variation on immune parameters), following the same standardisation procedure as in Visit 1. After resting for 10 minutes, blood and saliva samples will be collected. The ESWT will then be completed at the same speed as in Visit 1, lasting for 20 minutes. Within 5 minutes of exercise cessation, another blood and saliva sample will be collected. The participant will then rest for 1 hour, after which a final blood and saliva sample will be collected. This protocol is summarised in a schematic in Figure 1, and the outcome measures are explained in greater detail below.

The time delay between recruitment, visit 1 and visit 2 will be kept to a minimum in order to prevent deconditioning and minimise dropout rates. For dialysis patients, both study visits will be completed on a non-dialysis day that is not after their ‘long break’ (i.e. weekend, or two consecutive days without dialysis based on their regular shift pattern), in order to minimise the effect of fluid overload.

Physical performance

Endurance Capacity will be assessed using the ISWT (37) and ESWT (38). In the ISWT, the participant

For peer review only - http://bmjopen.bmj.com/site/about/guidelines.xhtml

walks a level 10m shuttle course at a speed controlled by an external audible bleep signal. The test progressively increases at 1 minute intervals for a total of 12 intervals, and is terminated when the participant fails to complete a shuttle within the required time. Following the ISWT, a walking speed equating to 85% of predicted peak O_2 consumption (VO_2peak) can be calculated, using a conversion table based upon their ISWT performance. The ESWT is completed at this continuous speed on the same shuttle course until volitional exhaustion or the end of the test (20 minutes) is reached. For the purposes of this study, any participant who does not complete the full 20 minutes of the ESWT in Visit 1 will be excluded to ensure standardisation of the test in Visit 2. This protocol was initially developed for use in patients with chronic airways obstruction (37) but has been used in the CKD population (39,40), showing good reproducibility and a high correlation with VO_2peak (41).

Venous blood sampling

Venous blood will be collected using venepuncture of the antecubital vein of either arm – provided the absence of an arteriovenous fistula. Blood will be drawn through a 21-guage needle (30 ml per time-point) and collected into K_2EDTA (di-potassium ethylenediaminetetraacetic acid), sodium citrate, sodium heparin and blank monovettes.

Blood processing, storage and analysis

Blood collected into EDTA tubes will be centrifuged at 4°C; the supernatant will then be aliquoted and frozen at -80ºC for future analysis. A cytometric bead array technique will be used to allow bulk-analysis of a panel of pro- and anti-inflammatory cytokines, including but not limited to IL-1, IL-2, IL-6, IL-10, TNF-α and IFNγ. Blood collected into sodium citrate (42) will be double-centrifuged at room temperature (15 mins at 2,500g, supernatant aliquoted followed by another 15 mins at 2,500g) to create platelet-poor plasma; the supernatant will then be aliquoted and frozen at -80ºC for future phenotyping of microparticles using flow cytometry, as explained below. Blood collected into sodium heparin will be analysed on the day of collection for immune cell subsets using flow cytometry, as explained below. Blood collected into blank monovettes will be sent to the Diagnostic Pathology Service at University Hospitals of Leicester NHS Trust on the day of collection for renal profile analysis, which includes eGFR, urea, bicarbonate, creatinine, sodium, potassium and phosphate measures. This will be completed for both patient and control populations.

Flow cytometry

Immune cells will be characterised based on their expression of surface antigens using flow cytometry. Monocyte subsets will be categorised as classical ($CD14^{++}CD16^{-}$), intermediate ($CD14^{++}CD16^{+}$) and non-classical ($CD14^{+}CD16^{++}$) (43). Monocytic ACE expression will be assessed using CD143, with IgG1 as a negative control. T cells will be categorised as helper ($CD3^{+}CD4^{+}$) cytotoxic ($CD3^{+}CD8^{+}$) and regulatory ($CD4^{+}CD25^{+}CD127^{-}$) T cells. B Cells will be identified as $CD3^{+}CD19^{+}$ and NK Cells as CD3-$CD56^{+}$. Following appropriate staining and washing procedures, immune cells will be analysed on a FACS Calibur (BD Biosciences, Oxford, UK). Acquisition templates and preliminary flow cytometry results are displayed in Figure 2.

Microparticles will be characterised based on their size, Annexin-v expression, cellular derivation and pro-thrombotic potential. Cellular derivations will be categorised as platelet-derived ($CD42b^{+}$), neutrophil-derived ($CD66b^{+}$), monocyte-derived ($CD14^{+}$) and endothelial cell-derived ($CD144^{+}$). Pro-thrombotic potential will be estimated using Tissue Factor ($CD142^{+}$) expression. Following the thawing of platelet-free-plasma at room temperature, samples will be double-centrifuged at room temperature (30 mins at 18,000g, supernatant removed, discarded and replaced with an equal volume of buffer, followed by another 30 mins at 18,000g). Samples will then undergo appropriate staining procedures with the antibodies listed above, after which microparticles will be analysed on a BD Accuri C6 (BD Biosciences, Oxford, UK) flow cytometer. Acquisition templates and preliminary

For peer review only - http://bmjopen.bmj.com/site/about/guidelines.xhtml

microparticle results are displayed in Figure 3. The protocol for isolating and analysing MPs is based upon a number of publications (42,44,45) and in our experience produces reliable results (Figure 3).

Saliva sampling and storage

Saliva samples will be collected into sterile plastic containers. Participants will swallow to empty the mouth, then open and hold the container themselves before performing a passive dribble of saliva collected under the tongue over the next two minutes. Following centrifugation, the supernatant will be aliquoted and frozen for future analysis, primarily for secretory Immunoglobulin A (sIgA) to investigate mucosal immunity.

Anthropometric measures

Height, weight and waist and hip circumference will be measured using standard procedures (46).

Body composition

Body composition parameters (i.e. fat and fat free mass content) will be measured using bioelectrical impedance analysis (BIA) (Inbody 370, Chicago, IL, USA). BIA has been validated for use in CKD (47). Whilst BIA has limitations with regards to reliability and is not recommended for tracking changes in body composotion, body composition will only be used as a descriptive rather than an outcome variable, and thus BIA has been deemed suitable to assess body composition for this reason.

Cardiovascular condition and function

Cardiac bioreactance analysis will be carried out using a Non-Invasive Cardiac Output Monitor (NICOM) (Cheetah Medical, Maidenhead, UK). Cardiac bioreactance is a quick, safe, validated (48), and non-invasive method of assessing central haemodynamics including heart rate, stroke volume, cardiac output, and total peripheral resistance. This is accomplished by using cutaneous electrodes placed on the chest in combination with an automatic sphygmomanometer to measure blood pressure.

Time spent in leisure activities

The Leisure Time Exercise Questionnaire (LTEQ) assesses the amount of exercise a patient undertakes, from which a metabolic equivalent (METS) estimation can be calculated. It also identifies preferred physical activities.

Fatigue

Fatigue is a common complaint CKD, and it has multiple potential mechanisms including anaemia (49) and cachexia (50). To assess self-reported fatigue, participants will be asked to fill in a fatigue scale (an internally designed Likert scale), as well as the validated Functional Assessment of Chronic Illness Therapy-Fatigue (FACIT-F) (51) which has been extensively used in CKD populations (52)

Muscle O_2 saturation

Reduced muscle O_2 saturation is another possible physiological mechanism of fatigue (53) and may be related to increased inflammation (54). A small non-invasive muscle O_2 saturation device (BSXInsight, BSXAthletics, TX, USA) will be fitted around the participant’s calf and worn during exercise bouts and subsequent recovery. This device uses near infrared spectroscopy (NIRS) to measure haemoglobin and myoglobin oxygenation (muscle O_2 tissue stores). NIRS has previously been used in a variety of disease conditions (55).

Clinical information

Clinical records will be used to extract information that allows associations to be made with physical activity levels and account for confounding variables, including: age; gender; ethnicity; primary cause

For peer review only - http://bmjopen.bmj.com/site/about/guidelines.xhtml

of renal failure; transplant or dialysis type; time since transplant or duration of dialysis; co-morbidities; and current medications.

Data analysis plan

Data analysis will compare groups at each time-point, investigate any changes elicited by exercise, and any other pertinent outcomes. Mixed-design ANOVAs will be used to analyse main effects of group and time and interaction effects, with Bonferroni post-hoc testing used to elucidate these effects. This will generate both tests of significance and estimates of effect sizes. Any non-normally distributed data will first be transformed appropriately to ensure normality assumptions are met, to increase statistical power and to allow clear conclusions to be drawn from the data. Where normality assumptions cannot be met via data transformation, Friedman's tests will be employed as a non-parametric alternative. This will allow the generation of hypotheses for future, larger studies.

Ethics and dissemination

Ethical and safety considerations

This protocol was reviewed by the East Midlands-Derby Research Ethics Committee (REC) and was given a favourable opinion (REC ref 15/EM/0391) on 24/09/2015. Additionally, local approval was given by the Research and Innovation office at the University Hospitals of Leicester NHS Trust (ref 11444) on 29/09/2015. Therefore, all steps have been taken when designing this protocol to minimise all ethical implications and ensure patient welfare. Two Substantial Amendments were made to this protocol, approved on 03/12/15 and 01/08/16 to add saliva collection, and fatigue questionnaires and muscle O_2 saturation respectively. The protocol presented here represents the most up to date version.

Dissemination plan

The results are planned for publication in early 2018. We plan to present the data at relevant national and international conferences, as well as publish the findings in relevant journals. Participant level data will be available at a later date.

Trial registration

This study is registered with the ISRCTN (ISRCTN38935454) The registration was completed after recruitment of the first patient, so the study was registered retrospectively.

Contributors: PJH wrote this manuscript; JN was involved in study design and protocol preparation; TJW was involved in study design and preparation of protocol amendments; NCB was involved in study design; ACS lead study design and protocol preparation. All authors reviewed this manuscript.

Funding: We are grateful to the Stoneygate Trust for partially funding this study. This work is supported by the National Institute for Health Research (NIHR) Diet, Lifestyle & Physical Activity Biomedical Research Unit based at University Hospitals of Leicester and Loughborough University. The views expressed are those of the authors and not necessarily those of the NHS, the NIHR or the Department of Health.

Competing interests: None declared.

Ethics Approval: REC East Midlands-Derby (ref 15/EM/0391).

References

1. Shlipak MG, Fried LF, Cushman M, Manolio T a, Peterson D, Stehman-Breen C, et al.

For peer review only - http://bmjopen.bmj.com/site/about/guidelines.xhtml

For peer review only

Cardiovascular mortality risk in chronic kidney disease: comparison of traditional and novel risk factors. JAMA. 2005;293(14):1737–45.

2. Caskey F, Castledine C, Dawnay a, Farrington K, Fogarty D, Fraser S, et al. UK Renal Registry 2015 18th Annual Report of the Renal Association. Nephron [Internet]. 2016;132(suppl1):1–366. Available from: www.renalreg.org

3. Johansen KL, Shubert T, Doyle J, Soher B, Sakkas GK, Kent-Braun J a. Muscle atrophy in patients receiving hemodialysis: effects on muscle strength, muscle quality, and physical function. Kidney Int [Internet]. 2002;63(1):291–7. Available from: http://www.nature.com/doifinder/10.1046/j.1523-1755.2003.00704.x\npapers3://publication/doi/10.1046/j.1523-1755.2003.00704.x

4. Fried LF, Lee JS, Shlipak M, Chertow GM, Green C, Ding J, et al. Chronic kidney disease and functional limitation in older people: health, aging and body composition study. J Am Geriatr Soc [Internet]. 2006;54(5):750–6. Available from: http://www.ncbi.nlm.nih.gov/pubmed/16696739

5. Fouque D, Kalantar-Zadeh K, Kopple J, Cano N, Chauveau P, Cuppari L, et al. A proposed nomenclature and diagnostic criteria for protein-energy wasting in acute and chronic kidney disease. Kidney Int [Internet]. Elsevier Masson SAS; 2008;73(4):391–8. Available from: http://www.ncbi.nlm.nih.gov/pubmed/18094682

6. Moreau K, Desseix A, Germain C, Barthe N, Bachelet T, Morel D, et al. Body composition in 98 patients awaiting kidney transplantation. Nutrition. 2014;30(2):186–91.

7. Johnson C, Gallagher-Lepak S, Zhu Y-R, Porth C, Kelber S, Roza A, et al. Factors influencing weight gain after renal transplantation. Transplantation. 1993;56(4):822–6.

8. Baum C. Weight Gain and Cardiovascular Risk After Organ Transplantation. J Parenter Enter Nutr [Internet]. 2001;25(3):114–9. Available from: http://www.thehastingscenter.org/Publications/BriefingBook/Detail.aspx?id=2198

9. van den Ham E, Kooman P, Leunissen KML, van Hooff JP. Posttransplantation weight gain is predominantly due to an increase in body fat mass. Transplantation. 2000;70(1):241–2.

10. Oberg BP, Mcmenamin E, Lucas FL, Mcmonagle E, Morrow J, Ikizler TA, et al. Increased prevalence of oxidant stress and inflammation in patients with moderate to severe chronic kidney disease. Kidney Int. 2004;65(3):1009–16.

11. Molnar MZ, Carrero JJ, Mucsi I, Remport A. Comparison of the malnutrition – inflammation score in chronic kidney disease patients and kidney transplant recipients. Int Urol Nephrol [Internet]. Springer Netherlands; 2015;1025–33. Available from: "http://dx.doi.org/10.1007/s11255-015-0984-2

12. Stenvinkel P, Heimbürger O, Paultre F, Diczfalusy U, Wang T, Berglund L, et al. Strong association between malnutrition, inflammation, and atherosclerosis in chronic renal failure. Kidney Int. 1999;55(5):1899–911.

13. Carrero JJ, Stenvinkel P, Cuppari L, Ikizler TA, Kalantar-Zadeh K, Kaysen G, et al. Etiology of the Protein-Energy Wasting Syndrome in Chronic Kidney Disease: A Consensus Statement From the International Society of Renal Nutrition and Metabolism (ISRNM). J Ren Nutr. 2013;23(2):77–90.

14. Hauser AB, Stinghen AEM, Kato S, Bucharles S, Aita C, Yuzawa Y, et al. Characteristics and causes of immune dysfunction related to uremia and dialysis. Perit Dial Int. 2008;28(SUPP. 3):183–7.

For peer review only - http://bmjopen.bmj.com/site/about/guidelines.xhtml

15. Girndt M, Sester U, Sester M, Kaul H, Köhler H. Impaired cellular immune function in patients with end-stage renal failure. Nephrol Dial Transplant. 1999;14(12):2807–10.

16. Hutchinson P, Chadban SJ, Atkins RC, Holdsworth SR. Laboratory assessment of immune function in renal transplant patients. Nephrol Dial Transplant [Internet]. 2003;18(5):983–9. Available from: http://ndt.oxfordjournals.org/cgi/doi/10.1093/ndt/gfg190

17. Vereyken EJF, Kraaij MD, Baan CC, Rezaee F, Weimar W, Wood KJ, et al. A shift towards pro-inflammatory CD16+ monocyte subsets with preserved cytokine production potential after kidney transplantation. PLoS One [Internet]. 2013;8(7):e70152. Available from: http://journals.plos.org/plosone/article?id=10.1371/journal.pone.0070152

18. Lu GY, Xu RJ, Zhang SH, Qiao Q, Shen L, Li M, et al. Alteration of circulatory platelet microparticles and endothelial microparticles in patients with chronic kidney disease. Int J Clin Exp Med. 2015;8(9):16704–8.

19. Vanwijk MJ, Vanbavel E, Sturk a, Nieuwland R. M icroparticles in cardiovascular diseases. Cardiovasc Res. 2003;59:277–87.

20. Stehr MD, Von Lengerke T. Preventing weight gain through exercise and physical activity in the elderly: A systematic review. Maturitas [Internet]. Elsevier Ireland Ltd; 2012;72(1):13–22. Available from: http://dx.doi.org/10.1016/j.maturitas.2012.01.022

21. Peterson MD, Sen A, Gordon PM. Influence of Resistance Exercise on Lean Body Mass in Aging Adults: A Meta-Analysis. Med Sci Sport Exerc. 2011;43(2):249–58.

22. de Vries NM, van Ravensberg CD, Hobbelen JSM, Olde Rikkert MGM, Staal JB, Nijhuis-van der Sanden MWG. Effects of physical exercise therapy on mobility, physical functioning, physical activity and quality of life in community-dwelling older adults with impaired mobility, physical disability and/or multi-morbidity: A meta-analysis. Ageing Res Rev [Internet]. Elsevier B.V.; 2012;11(1):136–49. Available from: http://dx.doi.org/10.1016/j.arr.2011.11.002

23. Chou C-H, Hwang C-L, Wu Y-T. Effect of Exercise on Physical Function, Daily Living Activities, and Quality of Life in the Frail Older Adults: A Meta-Analysis. Arch Phys Med Rehabil. 2012;93(2):237–44.

24. Woods J a., Vieira VJ, Keylock KT. Exercise, Inflammation, and Innate Immunity. Immunol Allergy Clin North Am. 2009;29(2):381–93.

25. Gleeson M, Bishop NC, Stensel DJ, Lindley MR, Mastana SS, Nimmo M a. The anti-inflammatory effects of exercise: mechanisms and implications for the prevention and treatment of disease. Nat Rev Immunol [Internet]. Nature Publishing Group; 2011;11(9):607–15. Available from: http://www.nature.com/doifinder/10.1038/nri3041

26. Nieman DC, Pedersen BK. Exercise and Immune Function. Sport Med. 1999;27(2):73–80.

27. Walsh NP, Gleeson M, Shephard RJ, Gleeson M, Woods JA, Bishop NC, et al. Position statement part one : immune function and exercise. Exerc Immunol Rev. 2011;17(1):6–63.

28. Babbitt DM, Diaz KM, Feairheller DL, Sturgeon KM, Perkins AM, Veerabhadrappa P, et al. Endothelial activation microparticles and inflammation status improve with exercise training in african americans. Int J Hypertens [Internet]. 2013;2013:538017. Available from: http://www.pubmedcentral.nih.gov/articlerender.fcgi?artid=3652180&tool=pmcentrez&rendertype=abstract

29. Wahl P, Jansen F, Achtzehn S, Schmitz T, Bloch W, Mester J, et al. Effects of High Intensity Training and High Volume Training on Endothelial Microparticles and Angiogenic Growth

For peer review only - http://bmjopen.bmj.com/site/about/guidelines.xhtml

Factors. PLoS One [Internet]. 2014;9(4):e96024. Available from: http://dx.plos.org/10.1371/journal.pone.0096024

30. Castaneda C, Layne JE, Munoz-Orians L, Gordon PL, Walsmith J, Foldvari M, et al. A Randomized Controlled Trial of Resistance Exercise Training to Improve Glycemic Control in Older Adults With Type 2 Diabetes. Diabetes Care. 2002;25(12):2335–41.

31. Mercer TH, Crawford C, Gleeson NP, Naish PF. Low-volume exercise rehabilitation improves functional capacity and self-reported functional status of dialysis patients. Am J Phys Med Rehabil [Internet]. 2002;81(3):162–7. Available from: http://www.ncbi.nlm.nih.gov/pubmed/11989511

32. Koufaki P, Mercer TH, Naish PF. Effects of exercise training on aerobic and functional capacity of end-stage renal disease patients. Clin Physiol Funct Imaging. 2002;22(2):115–24.

33. Mustata S, Groeneveld S, Davidson W, Ford G, Kiland K, Manns B. Effects of exercise training on physical impairment , arterial stiffness and health-related quality of life in patients with chronic kidney disease : a pilot study. Int Urol Nephrol. 2011;43(1):1133–41.

34. Viana JL, Kosmadakis GC, Watson EL, Bevington a., Feehally J, Bishop NC, et al. Evidence for Anti-Inflammatory Effects of Exercise in CKD. J Am Soc Nephrol [Internet]. 2014;25(9):2121–30. Available from: http://www.jasn.org/cgi/doi/10.1681/ASN.2013070702

35. Van Craenenbroeck AH, Van Ackeren K, Hoymans VY, Roeykens J, Verpooten G a., Vrints CJ, et al. Acute exercise-induced response of monocyte subtypes in chronic heart and renal failure. Mediators Inflamm. 2014;2014.

36. Johansen KL. Exercise and chronic kidney disease: current recommendations. Sport Med. 2005;35(6):485–99.

37. Singh SJ, Morgan MDL, Scott S, Walters D, Hardman AE. Development of a shuttle walking test of disability in patients with chronic airways obstruction. Thorax. 1992;47:1019–24.

38. Revill SM, Morgan MDL, Singh SJ, Williams J, Hardman a E. The endurance shuttle walk : a new field test for the assessment of endurance capacity in chronic obstructive pulmonary disease The endurance shuttle walk : a new field test for the assessment of endurance capacity in chronic obstructive pulmonary disease. Thorax. 1999;(July 2007):213–22.

39. Greenwood S a., Lindup H, Taylor K, Koufaki P, Rush R, MacDougall IC, et al. Evaluation of a pragmatic exercise rehabilitation programme in chronic kidney disease. Nephrol Dial Transplant. 2012;27(SUPPL. 3).

40. Wilund KR, Tomayko EJ, Wu P-T, Ryong Chung H, Vallurupalli S, Lakshminarayanan B, et al. Intradialytic exercise training reduces oxidative stress and epicardial fat: a pilot study. Nephrol Dial Transplant [Internet]. 2010;25(8):2695–701. Available from: http://ndt.oxfordjournals.org/cgi/doi/10.1093/ndt/gfq106

41. Painter P, Marcus RL. Assessing physical function and physical activity in patients with CKD. Clin J Am Soc Nephrol [Internet]. 2013;8(5):861–72. Available from: http://www.ncbi.nlm.nih.gov/pubmed/23220421

42. van der Heyde HC, Gramaglia I, Combes V, George TC, Grau GE. Flow Cytometric Analysis of Microparticles. Methods Mol Biol. 2011;699:337–54.

43. Ziegler-Heitbrock L, Hofer TPJ. Toward a Refined Definition of Monocyte Subsets. Front Immunol [Internet]. 2013;4(February):1–5. Available from: http://journal.frontiersin.org/article/10.3389/fimmu.2013.00023/abstract

For peer review only - http://bmjopen.bmj.com/site/about/guidelines.xhtml

For peer review only

44. Nielsen MH, Beck-Nielsen H, Andersen MN, Handberg A. A flow cytometric method for characterization of circulating cell-derived microparticles in plasma. J Extracell vesicles [Internet]. 2014;3:1–12. Available from: http://www.pubmedcentral.nih.gov/articlerender.fcgi?artid=3916676&tool=pmcentrez&rendertype=abstract

45. Jy W, Horstman L, Jiminez J, Ahn YS. Measuring circulating cell-derived microparticles. J Thromb Haemost. 2004;2(1):1842–51.

46. Eston R, Reilly T. Kinanthropometry and exercise physiology laboratory manual: anthropometry. 2009.

47. Chertow GM, Lowrie EG, Wilmore DW, Gonzalez J, Lew NL, Ling J, et al. Nutritional Assessment with Bioelectrical Impedance Analysis in Maintenance Hemodialysis Patients. J Am Soc Nephrol. 1995;6:75–81.

48. Squara P, Denjean D, Estagnasie P, Brusset A, Dib JC, Dubois C. Noninvasive cardiac output monitoring (NICOM): a clinical validation. Intensive Care Med. 2007;33:1191–4.

49. Obrador GT, Pereira BJG. Anaemia of chronic kidney disease : an under-recognized and under-treated problem. Nephrol Dial Transplant. 2002;177(S11):44–6.

50. Cheung WW, Paik KH, Mak RH. Inflammation and cachexia in chronic kidney disease. Pediatr Nephrol [Internet]. 2010;25(4):711–24. Available from: http://link.springer.com/10.1007/s00467-009-1427-z

51. Chao C-T, Huang J-W, Chiang C-K. Functional assessment of chronic illness therapy - the fatigue scale exhibits stronger associations with clinical parameters in chronic dialysis patients compared to other fatigue-assessing instruments. PeerJ. 2016;1818.

52. Jhamb M, Yabes J, Steel L, Amanda M, Unruh M. Prevalence and Correlates of Fatigue in Chronic Kidney Disease and End-Stage Renal Disease : Are Sleep Disorders a Key to Understanding Fatigue ? Am J Nephrol. 2013;38:489–95.

53. Gardner AW, Parker DE, Webb N, Montgomery PS, Scott KJ, Blevins SM, et al. Calf muscle hemoglobin oxygen saturation characteristics and exercise performance in patients with intermittent claudication. J Vasc Surg. 2008;48(3):644–9.

54. Wust RCI, Degens H. Factors contributing to muscle wasting and dysfunction in COPD patients. Int J COPD. 2007;2(3):289–300.

55. Grassi B, Quaresima V. Near-infrared spectroscopy and skeletal muscle oxidative function *in vivo* in health and disease: a review from an exercise physiology perspective. J Biomed Opt [Internet]. 2016;21(9):91313. Available from: http://biomedicaloptics.spiedigitallibrary.org/article.aspx?doi=10.1117/1.JBO.21.9.091313

For peer review only

For peer review only - http://bmjopen.bmj.com/site/about/guidelines.xhtml

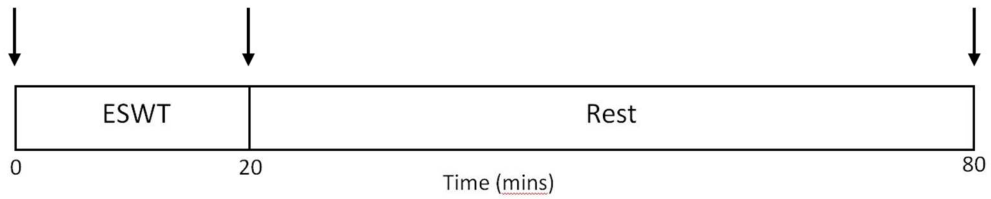

Figure 1. Visit 2 exercise and sample collection protocol. ↓= Venous blood and saliva collection. ESWT = Endurance Shuttle Walk Test

111x23mm (300 x 300 DPI)

For peer review only

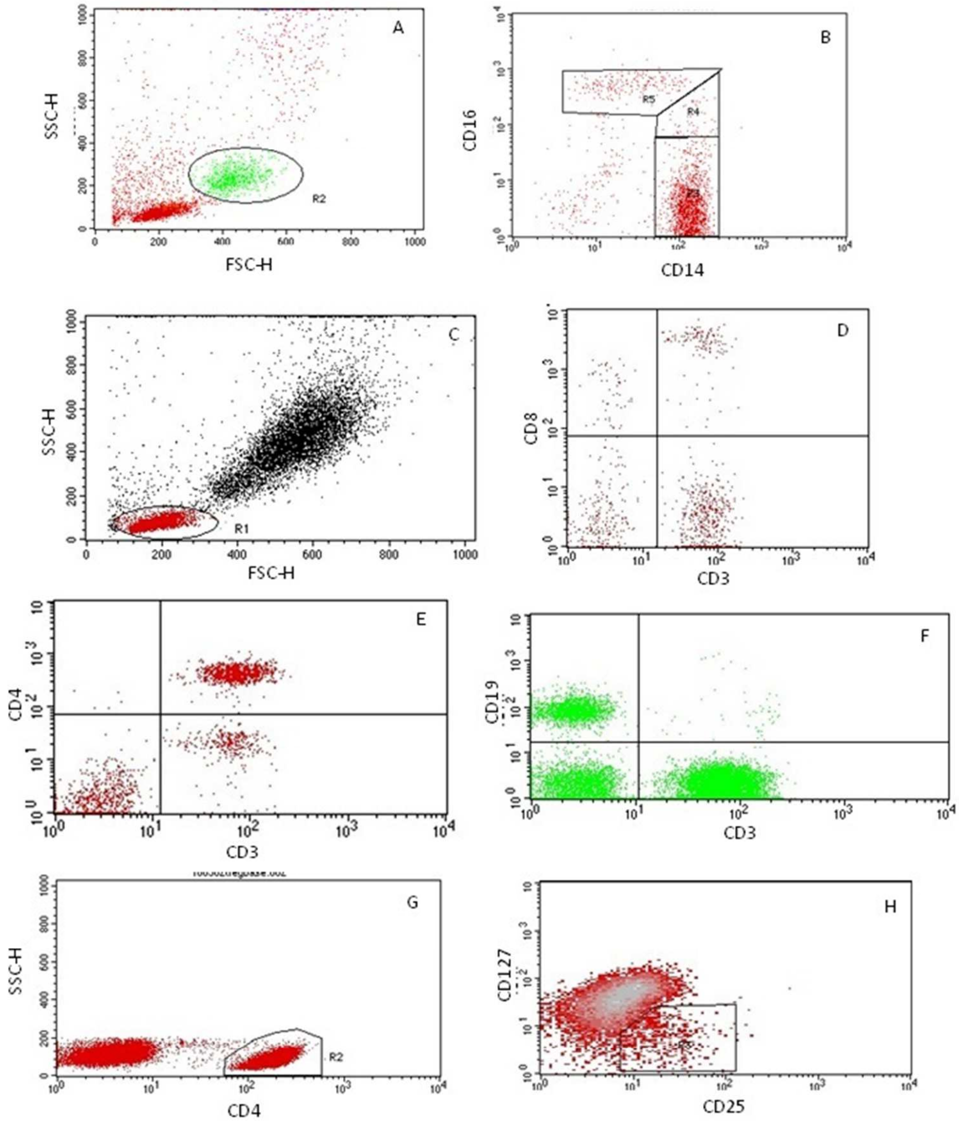

Figure 2. Demonstration of the gating strategy used to identify immune cell subsets. A: Total monocyte gating. B: Monocyte subsets (R3 = classical (CD14++CD16-), R4 = intermediate (CD14++CD16+), R5 = non-classical (CD14+CD16++). C: Total lymphocyte gating. D: Gating CD8+ cytotoxic T lymphocytes in the upper right quadrant. E: Gating CD4+ helper T lymphocytes in the upper right quadrant. F: Gating B lymphocytes (CD3-CD19+) in the upper left quadrant. G:Initial T-Reg gating, identifying CD4+ lymphocytes. H: Secondary T-reg gating, back-gated onto plot G, further identifying the CD4+ lymphocytes that are CD25+CD127-. Not all graphs display 100% of acquired cells – this has been altered independently to allow ease of gating.

60x72mm (300 x 300 DPI)

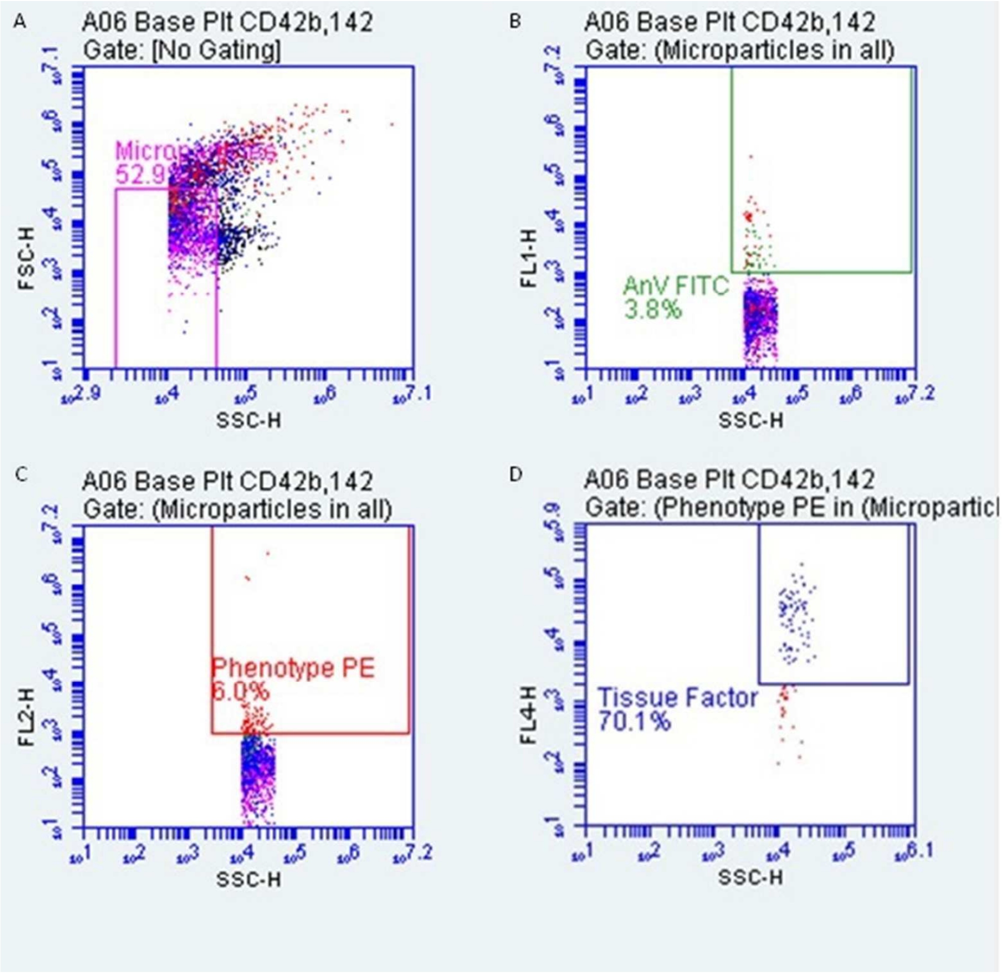

Figure 3. Demonstration of the gating strategy used to characterise microparticles. A: 'All microparticles', set using beads of known size. B: 'All microparticles' based on Annexin-v expression. C: Phenotype marker expression, used to quantify microparticles of different cellular sources. D: Pro-thrombotic potential, assessed by Tissue Factor expression. All gates have been appropriately sized and positioned using unstained samples to distinguish negative vs. positive staining.

52x51mm (300 x 300 DPI)

Physical Activity, Immune Function and Inflammation in Kidney Patients (the PINK Study): A Feasibility Trial Protocol

Journal:	*BMJ Open*
Manuscript ID	bmjopen-2016-014713.R3
Article Type:	Protocol
Date Submitted by the Author:	21-Mar-2017
Complete List of Authors:	Highton, Patrick; Loughborough University, School of Sport, Exercise and Health Sciences; University of Leicester, Department of Infection, Immunity and Inflammation<br>Neale, Jill; University of Leicester, Department of Infection, Immunity and Inflammation<br>Wilkinson, Thomas; University of Leicester, Department of Infection, Immunity and Inflammation<br>Bishop , Nicolette ; Loughborough University, School of Sport, Exercise and Health Sciences; University of Leicester, Department of Infection, Immunity and Inflammation<br>Smith, Alice; University of Leicester, Department of Infection, Immunity and Inflammation; Loughborough University, School of Sport, Exercise and Health Sciences
**Primary Subject Heading**:	Immunology (including allergy)
Secondary Subject Heading:	Sports and exercise medicine, Renal medicine
Keywords:	Renal transplantation < NEPHROLOGY, IMMUNOLOGY, Inflammation, Exercise

Physical Activity, Immune Function and Inflammation in Kidney Patients (the PINK Study): A Feasibility Trial Protocol

Patrick J Highton[1,2], Jill Neale[2], Thomas J Wilkinson[2], Nicolette C Bishop[1,2], Alice C Smith[1,2].

Corresponding author:

Patrick J Highton
Leicester Kidney Exercise Team
Academic Unit
Leicester General Hospital
LE5 4PW
P.Highton@lboro.ac.uk
0116 258 4346

[1] School of Sport, Exercise and Health Sciences, Loughborough University, Loughborough, United Kingdom

[2] Department of Infection, Immunity and Inflammation, University of Leicester, Leicester, United Kingdom

Keywords: renal transplantation, nephrology, immunology, inflammation, exercise

Word count (excluding title page, abstract, references, figures and tables): 2523

For peer review only

Abstract

Introduction: Chronic Kidney Disease (CKD) patients display increased infection-related mortality and elevated cardiovascular risk only partly attributed to traditional risk factors. CKD patients also exhibit a pro-inflammatory environment and impaired immune function. Aerobic exercise has the potential to positively impact these detriments, but is under-researched in this patient population. This feasibility study will investigate the effects of acute aerobic exercise on inflammation and immune function in CKD patients, to inform the design of larger studies intended to ultimately influence current exercise recommendations.

Methods and analysis: Patients with CKD, including renal transplant recipients, will visit the laboratory on two occasions, both preceded by appropriate exercise, alcohol and caffeine restrictions. On Visit 1, baseline assessments will be completed, comprising anthropometrics, body composition, cardiovascular function, and fatigue and leisure time exercise questionnaires. Participants will then undertake an incremental shuttle walk test, to estimate VO_2peak. On Visit 2, participants will complete a 20 minute shuttle walk at a constant speed to achieve 85% estimated VO_2peak. Blood and saliva samples will be taken before, immediately after and 1 hour after this exercise bout. Muscle O_2 saturation will be monitored throughout exercise and recovery. Age and sex-matched non-CKD 'healthy control' participants will complete an identical protocol. Blood and saliva samples will be analysed for markers of inflammation and immune function, using cytometric bead array and flow cytometry techniques. Appropriate statistical tests will be used to analyse the data.

Ethics and dissemination: A favourable opinion was granted by the East Midlands-Derby Research Ethics Committee on 18/09/2015 (ref 15/EM/0391) and the study was approved and sponsored by University Hospitals of Leicester Research and Innovation (ref 11444). The study was registered with ISRCTN (ref 38935454). The results will be presented at relevant conferences, and it is anticipated that the reports will be published in appropriate journals in 2018.

Strengths and limitations of this study
• Inclusion of control group matched for age and sex<br>• A variety of outcome measures to inform future study design<br>• Pragmatic study design which will inform future exercise recommendations for patients with CKD<br>• No non-exercise control visit

Introduction

Patients with all stages of Chronic Kidney Disease (CKD) have elevated cardiovascular disease (CVD) risk that cannot be fully explained by traditional risk factors (1). CVD is the most common cause of death amongst CKD patients (23% in 2013), followed by infection (19% in 2013) (2).

CKD patients are often sarcopenic and obese (3–6), and further deterioration in these characteristics is often observed after renal transplantation (7–9). Patients with CKD and renal transplant recipients (RTRs) also frequently suffer from chronic systemic inflammation (10,11), which can worsen cachexia and increase cardiovascular risk in CKD (12,13). CKD patients and RTRs also display impaired cellular immune function (14–16), which may explain why infection is the second-leading cause of death in this population (2). This effect (i.e. impaired immune function) is further compounded in RTRs by the immunosuppressive drug regime. Impaired immune function may

For peer review only - http://bmjopen.bmj.com/site/about/guidelines.xhtml

further exacerbate inflammation due to alterations in circulating immune cell subsets (17) which could serve to worsen body composition and increase cardiovascular risk. Finally, CKD patients display elevated levels of circulating pro-thrombotic microparticles (MPs) (18), which may worsen CVD risk and/or burden (19).

Exercise has the potential to benefit and improve many of the aforementioned inter-related morbidities. In the general population, exercise can positively impact upon weight gain (20), muscle wasting (21), physical capacity (22) and fatigue (23). Further, moderate aerobic exercise can also modify systemic inflammation (24,25) bolster immune function via alterations in circulating immune cell populations and activity, and reduce circulating MP levels (26–29).

In the CKD population, similar positive effects of exercise on body composition, physical function and quality of life have been demonstrated (30–33), however the research on inflammation and immune function in this population is limited. Previous research has shown that 30 minutes of moderate intensity walking exercise elicited a normal pattern of leukocyte and monocyte activation, whilst enhancing granulocyte function and promoting an anti-inflammatory environment (increased Interleukin-10 release) in pre-dialysis CKD patients (34). However, in a similar population an exhaustive bout of cycling exercise elicited a shift towards the more pro-inflammatory $CD16^+$ monocyte, potentially favouring inflammation (35). This disparity may be due to the exercise intensities investigated – moderate intensity exercise is more beneficial in strengthening immune function and preventing infection (26) and is promoted in general exercise guidelines. As such, investigating the effects of moderate intensity exercise in this patient population is more pragmatic and will help to guide future exercise recommendations, which are lacking in this patient population.

Therefore, this feasibility study aims investigate the effects of acute aerobic walking exercise on the inflammation and immune function in CKD patients. This will create the basis from which a larger trial can be conducted, including data for a power calculation, which will ultimately help to inform exercise recommendations in this population which are currently lacking, particularly with regards to immune function and inflammation (36). However, a preliminary power calculation (GPower 3.1) based upon the findings of Viana et al (34) concerning total lymphocyte count (based on a statistical power of 0.80 and an alpha level of 0.05) suggests that 15 participants per group (e.g. pre-dialysis CKD, renal transplant recipients, healthy controls) will be sufficient.

Methods and Analysis

Outcomes to be measured

This study will investigate the effect of 20 minutes of moderate intensity walking exercise on immune cell subsets and inflammatory markers. Participants will be grouped based on their status (i.e. 'patient' or 'healthy control'). Both groups will complete an identical acute, cross-sectional study protocol necessitating two study visits, as explained below.

Participant recruitment

All CKD patients attending outpatient clinics within the University Hospitals of Leicester renal network will be screened for eligibility by their consultant nephrologist prior to recruitment, and approached during their routine outpatient appointments. The inclusion and exclusion criteria for patients are summarised in Table 1. Healthy age and sex matched controls with no known chronic disease will be recruited from the local community. Those who do not believe themselves to suffer from any significant chronic disease will be eligible to participate. The 'broad' inclusion and exclusion criteria allows the incorporation of several different groups (i.e. non-dialysis CKD patients, dialysis

For peer review only - http://bmjopen.bmj.com/site/about/guidelines.xhtml

patients, renal transplant recipients and healthy controls), and thus will allow comparison between these groups. Similarly, the wide range of CKD stage will allow the influence of remaining renal function on the measured markers to be investigated.

Inclusion Criteria	Exclusion Criteria
▪ Established chronic kidney disease (all stages will be eligible including those with an established kidney transplant and those receiving dialysis treatment)	▪ Age under 18 years<br>▪ Pregnancy<br>▪ Received kidney transplant less than 6 months prior to study entry<br>▪ Any element of study assessment protocol considered by principle care provider to be contraindicated due to physical impairment, comorbidity or any other reason<br>▪ Inability to give informed consent for any reason<br>▪ Visual or hearing impairment or insufficient command of English to give informed consent or comply with the assessment protocol

Table 1. Inclusion and exclusion criteria for patients.

Trial design and timeline

This is a non-randomised, controlled, feasibility study. Participants will complete two study visits as described below.

Visit 1: Participants will arrive at the laboratory in the morning, unfasted but having consumed no caffeine or alcohol and completed no strenuous exercise for 24 hours. Participants will complete questionnaires about time spent in leisure activity and their perception of fatigue, and assessments of anthropometry, body composition and cardiovascular condition. Participants will then undertake the Incremental Shuttle Walk Test (ISWT), followed by the Endurance Shuttle Walk Test (ESWT) as explained below. If the participant cannot complete the full 20 minute duration of the ESWT, they will be withdrawn from the study.

Visit 2: Participants will arrive at the laboratory in the morning (8-10am, to minimise the influence of diurnal variation on immune parameters), following the same standardisation procedure as in Visit 1. After resting for 10 minutes, blood and saliva samples will be collected. The ESWT will then be completed at the same speed as in Visit 1, lasting for 20 minutes. Within 5 minutes of exercise cessation, another blood and saliva sample will be collected. The participant will then rest for 1 hour, after which a final blood and saliva sample will be collected. This protocol is summarised in a schematic in Figure 1, and the outcome measures are explained in greater detail below.

The time delay between recruitment, visit 1 and visit 2 will be kept to a minimum in order to prevent deconditioning and minimise dropout rates. For dialysis patients, both study visits will be completed on a non-dialysis day that is not after their ‘long break’ (i.e. weekend, or two consecutive days without dialysis based on their regular shift pattern), in order to minimise the effect of fluid overload.

Physical performance

Endurance Capacity will be assessed using the ISWT (37) and ESWT (38). In the ISWT, the participant

For peer review only - http://bmjopen.bmj.com/site/about/guidelines.xhtml

walks a level 10m shuttle course at a speed controlled by an external audible bleep signal. The test progressively increases at 1 minute intervals for a total of 12 intervals, and is terminated when the participant fails to complete a shuttle within the required time. Following the ISWT, a walking speed equating to 85% of predicted peak O_2 consumption (VO_2peak) can be calculated, using a conversion table based upon their ISWT performance. The ESWT is completed at this continuous speed on the same shuttle course until volitional exhaustion or the end of the test (20 minutes) is reached. For the purposes of this study, any participant who does not complete the full 20 minutes of the ESWT in Visit 1 will be excluded to ensure standardisation of the test in Visit 2. This protocol was initially developed for use in patients with chronic airways obstruction (37) but has been used in the CKD population (39,40), showing good reproducibility and a high correlation with VO_2peak (41).

Venous blood sampling

Venous blood will be collected using venepuncture of the antecubital vein of either arm – provided the absence of an arteriovenous fistula. Blood will be drawn through a 21-guage needle (30 ml per time-point) and collected into K_2EDTA (di-potassium ethylenediaminetetraacetic acid), sodium citrate, sodium heparin and blank monovettes.

Blood processing, storage and analysis

Blood collected into EDTA tubes will be centrifuged at 4°C; the supernatant will then be aliquoted and frozen at -80°C for future analysis. A cytometric bead array technique will be used to allow bulk-analysis of a panel of pro- and anti-inflammatory cytokines, including but not limited to IL-1, IL-2, IL-6, IL-10, TNF-α and IFNγ. Blood collected into sodium citrate (42) will be double-centrifuged at room temperature (15 mins at 2,500g, supernatant aliquoted followed by another 15 mins at 2,500g) to create platelet-poor plasma; the supernatant will then be aliquoted and frozen at -80°C for future phenotyping of microparticles using flow cytometry, as explained below. Blood collected into sodium heparin will be analysed on the day of collection for immune cell subsets using flow cytometry, as explained below. Blood collected into blank monovettes will be sent to the Diagnostic Pathology Service at University Hospitals of Leicester NHS Trust on the day of collection for renal profile analysis, which includes eGFR, urea, bicarbonate, creatinine, sodium, potassium and phosphate measures. This will be completed for both patient and control populations.

Flow cytometry

Immune cells will be characterised based on their expression of surface antigens using flow cytometry. Monocyte subsets will be categorised as classical ($CD14^{++}CD16^{-}$), intermediate ($CD14^{++}CD16^{+}$) and non-classical ($CD14^{+}CD16^{++}$) (43). Monocyte Angiotensin-Converting Enzyme expression will be assessed using CD143, with IgG1 as a negative control. T cells will be categorised as helper ($CD3^{+}CD4^{+}$) cytotoxic ($CD3^{+}CD8^{+}$) and regulatory ($CD4^{+}CD25^{+}CD127^{-}$) T cells. B Cells will be identified as $CD3^{+}CD19^{+}$ and NK Cells as CD3-$CD56^{+}$. Following appropriate staining and washing procedures, immune cells will be analysed on a FACS Calibur (BD Biosciences, Oxford, UK). Acquisition templates and preliminary flow cytometry results are displayed in Figure 2.

Microparticles will be characterised based on their size, Annexin-v expression, cellular derivation and pro-thrombotic potential. Cellular derivations will be categorised as platelet-derived ($CD42b^{+}$), neutrophil-derived ($CD66b^{+}$), monocyte-derived ($CD14^{+}$) and endothelial cell-derived ($CD144^{+}$). Pro-thrombotic potential will be estimated using Tissue Factor ($CD142^{+}$) expression. Following the thawing of platelet-free-plasma at room temperature, samples will be double-centrifuged at room temperature (30 mins at 18,000g, supernatant removed, discarded and replaced with an equal volume of buffer, followed by another 30 mins at 18,000g). Samples will then undergo appropriate staining procedures with the antibodies listed above, after which microparticles will be analysed on a BD Accuri C6 (BD Biosciences, Oxford, UK) flow cytometer. Acquisition templates and preliminary

For peer review only - http://bmjopen.bmj.com/site/about/guidelines.xhtml

microparticle results are displayed in Figure 3. The protocol for isolating and analysing MPs is based upon a number of publications (42,44,45) and in our experience produces reliable results (Figure 3).

Saliva sampling and storage

Saliva samples will be collected into sterile plastic containers. Participants will swallow to empty the mouth, then open and hold the container themselves before performing a passive dribble of saliva collected under the tongue over the next two minutes. Following centrifugation, the supernatant will be aliquoted and frozen for future analysis, primarily for secretory Immunoglobulin A (sIgA) to investigate mucosal immunity.

Anthropometric measures

Height, weight and waist and hip circumference will be measured using standard procedures (46).

Body composition

Body composition parameters (i.e. fat and fat free mass content) will be measured using bioelectrical impedance analysis (BIA) (Inbody 370, Chicago, IL, USA). BIA has been validated for use in CKD (47). Whilst BIA has limitations with regards to reliability and is not recommended for tracking changes in body composition, body composition will only be used as a descriptive rather than an outcome variable, and thus BIA has been deemed suitable to assess body composition for this reason.

Cardiovascular condition and function

Cardiac bioreactance analysis will be carried out using a Non-Invasive Cardiac Output Monitor (NICOM) (Cheetah Medical, Maidenhead, UK). Cardiac bioreactance is a quick, safe, validated (48), and non-invasive method of assessing central haemodynamics including heart rate, stroke volume, cardiac output, and total peripheral resistance. This is accomplished by using cutaneous electrodes placed on the chest in combination with an automatic sphygmomanometer to measure blood pressure.

Time spent in leisure activities

The Leisure Time Exercise Questionnaire (LTEQ) assesses the amount of exercise a patient undertakes, from which a metabolic equivalent (METS) estimation can be calculated. It also identifies preferred physical activities.

Fatigue

Fatigue is a common complaint CKD, and it has multiple potential mechanisms including anaemia (49) and cachexia (50). To assess self-reported fatigue, participants will be asked to fill in a fatigue scale (an internally designed Likert scale), as well as the validated Functional Assessment of Chronic Illness Therapy-Fatigue (FACIT-F) (51) which has been extensively used in CKD populations (52)

Muscle O_2 saturation

Reduced muscle O_2 saturation is another possible physiological mechanism of fatigue (53) and may be related to increased inflammation (54). A small non-invasive muscle O_2 saturation device (BSXInsight, BSXAthletics, TX, USA) will be fitted around the participant's calf and worn during exercise bouts and subsequent recovery. This device uses near infrared spectroscopy (NIRS) to measure haemoglobin and myoglobin oxygenation (muscle O_2 tissue stores). NIRS has previously been used in a variety of disease conditions (55).

Clinical information

Clinical records will be used to extract information that allows associations to be made with physical activity levels and account for confounding variables, including: age; gender; ethnicity; primary cause

For peer review only - http://bmjopen.bmj.com/site/about/guidelines.xhtml

For peer review only

of renal failure; transplant or dialysis type; time since transplant or duration of dialysis; co-morbidities; and current medications.

Data analysis plan

Mixed-design ANOVAs will be used to analyse main effects of group and time and interaction effects, with Bonferroni post-hoc testing used to elucidate these effects. This will generate both tests of significance and estimates of effect sizes. Any non-normally distributed data will first be transformed appropriately to ensure normality assumptions are met, to increase statistical power and to allow clear conclusions to be drawn from the data. Where normality assumptions cannot be met via data transformation, Friedman's tests will be employed as a non-parametric alternative. This will allow the generation of hypotheses for future, larger studies.

Ethics and dissemination

Ethical and safety considerations

This protocol was reviewed by the East Midlands-Derby Research Ethics Committee (REC) and was given a favourable opinion (REC ref 15/EM/0391) on 24/09/2015. Additionally, local approval was given by the Research and Innovation office at the University Hospitals of Leicester NHS Trust (ref 11444) on 29/09/2015. Therefore, all steps have been taken when designing this protocol to minimise all ethical implications and ensure patient welfare. Two Substantial Amendments were made to this protocol, approved on 03/12/15 and 01/08/16 to add saliva collection, and fatigue questionnaires and muscle O_2 saturation respectively. The protocol presented here represents the most up to date version.

Dissemination plan

The results are planned for publication in early 2018. We plan to present the data at relevant national and international conferences, as well as publish the findings in relevant journals. Participant level data will be available at a later date.

Trial registration

This study is registered with the ISRCTN (ISRCTN38935454) The registration was completed after recruitment of the first patient, so the study was registered retrospectively.

Contributors: PJH wrote this manuscript; JN was involved in study design and protocol preparation; TJW was involved in study design and preparation of protocol amendments; NCB was involved in study design; ACS lead study design and protocol preparation. All authors reviewed this manuscript.

Funding: We are grateful to the Stoneygate Trust for partially funding this study. This work is supported by the National Institute for Health Research (NIHR) Diet, Lifestyle & Physical Activity Biomedical Research Unit based at University Hospitals of Leicester and Loughborough University. The views expressed are those of the authors and not necessarily those of the NHS, the NIHR or the Department of Health.

Competing interests: None declared.

Ethics Approval: REC East Midlands-Derby (ref 15/EM/0391).

References

1. Shlipak MG, Fried LF, Cushman M, Manolio T a, Peterson D, Stehman-Breen C, et al. Cardiovascular mortality risk in chronic kidney disease: comparison of traditional and novel

For peer review only - http://bmjopen.bmj.com/site/about/guidelines.xhtml

risk factors. JAMA. 2005;293(14):1737–45.

2. Caskey F, Castledine C, Dawnay a, Farrington K, Fogarty D, Fraser S, et al. UK Renal Registry 2015 18th Annual Report of the Renal Association. Nephron [Internet]. 2016;132(suppl1):1–366. Available from: www.renalreg.org

3. Johansen KL, Shubert T, Doyle J, Soher B, Sakkas GK, Kent-Braun J a. Muscle atrophy in patients receiving hemodialysis: effects on muscle strength, muscle quality, and physical function. Kidney Int [Internet]. 2002;63(1):291–7. Available from: http://www.nature.com/doifinder/10.1046/j.1523-1755.2003.00704.x\npapers3://publication/doi/10.1046/j.1523-1755.2003.00704.x

4. Fried LF, Lee JS, Shlipak M, Chertow GM, Green C, Ding J, et al. Chronic kidney disease and functional limitation in older people: health, aging and body composition study. J Am Geriatr Soc [Internet]. 2006;54(5):750–6. Available from: http://www.ncbi.nlm.nih.gov/pubmed/16696739

5. Fouque D, Kalantar-Zadeh K, Kopple J, Cano N, Chauveau P, Cuppari L, et al. A proposed nomenclature and diagnostic criteria for protein-energy wasting in acute and chronic kidney disease. Kidney Int [Internet]. Elsevier Masson SAS; 2008;73(4):391–8. Available from: http://www.ncbi.nlm.nih.gov/pubmed/18094682

6. Moreau K, Desseix A, Germain C, Barthe N, Bachelet T, Morel D, et al. Body composition in 98 patients awaiting kidney transplantation. Nutrition. 2014;30(2):186–91.

7. Johnson C, Gallagher-Lepak S, Zhu Y-R, Porth C, Kelber S, Roza A, et al. Factors influencing weight gain after renal transplantation. Transplantation. 1993;56(4):822–6.

8. Baum C. Weight Gain and Cardiovascular Risk After Organ Transplantation. J Parenter Enter Nutr [Internet]. 2001;25(3):114–9. Available from: http://www.thehastingscenter.org/Publications/BriefingBook/Detail.aspx?id=2198

9. van den Ham E, Kooman P, Leunissen KML, van Hooff JP. Posttransplantation weight gain is predominantly due to an increase in body fat mass. Transplantation. 2000;70(1):241–2.

10. Oberg BP, Mcmenamin E, Lucas FL, Mcmonagle E, Morrow J, Ikizler TA, et al. Increased prevalence of oxidant stress and inflammation in patients with moderate to severe chronic kidney disease. Kidney Int. 2004;65(3):1009–16.

11. Molnar MZ, Carrero JJ, Mucsi I, Remport A. Comparison of the malnutrition – inflammation score in chronic kidney disease patients and kidney transplant recipients. Int Urol Nephrol [Internet]. Springer Netherlands; 2015;1025–33. Available from: "http://dx.doi.org/10.1007/s11255-015-0984-2

12. Stenvinkel P, Heimbürger O, Paultre F, Diczfalusy U, Wang T, Berglund L, et al. Strong association between malnutrition, inflammation, and atherosclerosis in chronic renal failure. Kidney Int. 1999;55(5):1899–911.

13. Carrero JJ, Stenvinkel P, Cuppari L, Ikizler TA, Kalantar-Zadeh K, Kaysen G, et al. Etiology of the Protein-Energy Wasting Syndrome in Chronic Kidney Disease: A Consensus Statement From the International Society of Renal Nutrition and Metabolism (ISRNM). J Ren Nutr. 2013;23(2):77–90.

14. Hauser AB, Stinghen AEM, Kato S, Bucharles S, Aita C, Yuzawa Y, et al. Characteristics and causes of immune dysfunction related to uremia and dialysis. Perit Dial Int. 2008;28(SUPP. 3):183–7.

For peer review only - http://bmjopen.bmj.com/site/about/guidelines.xhtml

For peer review only

15. Girndt M, Sester U, Sester M, Kaul H, Köhler H. Impaired cellular immune function in patients with end-stage renal failure. Nephrol Dial Transplant. 1999;14(12):2807–10.

16. Hutchinson P, Chadban SJ, Atkins RC, Holdsworth SR. Laboratory assessment of immune function in renal transplant patients. Nephrol Dial Transplant [Internet]. 2003;18(5):983–9. Available from: http://ndt.oxfordjournals.org/cgi/doi/10.1093/ndt/gfg190

17. Vereyken EJF, Kraaij MD, Baan CC, Rezaee F, Weimar W, Wood KJ, et al. A shift towards pro-inflammatory CD16+ monocyte subsets with preserved cytokine production potential after kidney transplantation. PLoS One [Internet]. 2013;8(7):e70152. Available from: http://journals.plos.org/plosone/article?id=10.1371/journal.pone.0070152

18. Lu GY, Xu RJ, Zhang SH, Qiao Q, Shen L, Li M, et al. Alteration of circulatory platelet microparticles and endothelial microparticles in patients with chronic kidney disease. Int J Clin Exp Med. 2015;8(9):16704–8.

19. Vanwijk MJ, Vanbavel E, Sturk a, Nieuwland R. M icroparticles in cardiovascular diseases. Cardiovasc Res. 2003;59:277–87.

20. Stehr MD, Von Lengerke T. Preventing weight gain through exercise and physical activity in the elderly: A systematic review. Maturitas [Internet]. Elsevier Ireland Ltd; 2012;72(1):13–22. Available from: http://dx.doi.org/10.1016/j.maturitas.2012.01.022

21. Peterson MD, Sen A, Gordon PM. Influence of Resistance Exercise on Lean Body Mass in Aging Adults: A Meta-Analysis. Med Sci Sport Exerc. 2011;43(2):249–58.

22. de Vries NM, van Ravensberg CD, Hobbelen JSM, Olde Rikkert MGM, Staal JB, Nijhuis-van der Sanden MWG. Effects of physical exercise therapy on mobility, physical functioning, physical activity and quality of life in community-dwelling older adults with impaired mobility, physical disability and/or multi-morbidity: A meta-analysis. Ageing Res Rev [Internet]. Elsevier B.V.; 2012;11(1):136–49. Available from: http://dx.doi.org/10.1016/j.arr.2011.11.002

23. Chou C-H, Hwang C-L, Wu Y-T. Effect of Exercise on Physical Function, Daily Living Activities, and Quality of Life in the Frail Older Adults: A Meta-Analysis. Arch Phys Med Rehabil. 2012;93(2):237–44.

24. Woods J a., Vieira VJ, Keylock KT. Exercise, Inflammation, and Innate Immunity. Immunol Allergy Clin North Am. 2009;29(2):381–93.

25. Gleeson M, Bishop NC, Stensel DJ, Lindley MR, Mastana SS, Nimmo M a. The anti-inflammatory effects of exercise: mechanisms and implications for the prevention and treatment of disease. Nat Rev Immunol [Internet]. Nature Publishing Group; 2011;11(9):607–15. Available from: http://www.nature.com/doifinder/10.1038/nri3041

26. Nieman DC, Pedersen BK. Exercise and Immune Function. Sport Med. 1999;27(2):73–80.

27. Walsh NP, Gleeson M, Shephard RJ, Gleeson M, Woods JA, Bishop NC, et al. Position statement part one : immune function and exercise. Exerc Immunol Rev. 2011;17(1):6–63.

28. Babbitt DM, Diaz KM, Feairheller DL, Sturgeon KM, Perkins AM, Veerabhadrappa P, et al. Endothelial activation microparticles and inflammation status improve with exercise training in african americans. Int J Hypertens [Internet]. 2013;2013:538017. Available from: http://www.pubmedcentral.nih.gov/articlerender.fcgi?artid=3652180&tool=pmcentrez&rendertype=abstract

29. Wahl P, Jansen F, Achtzehn S, Schmitz T, Bloch W, Mester J, et al. Effects of High Intensity Training and High Volume Training on Endothelial Microparticles and Angiogenic Growth

For peer review only - http://bmjopen.bmj.com/site/about/guidelines.xhtml

Factors. PLoS One [Internet]. 2014;9(4):e96024. Available from: http://dx.plos.org/10.1371/journal.pone.0096024

30. Castaneda C, Layne JE, Munoz-Orians L, Gordon PL, Walsmith J, Foldvari M, et al. A Randomized Controlled Trial of Resistance Exercise Training to Improve Glycemic Control in Older Adults With Type 2 Diabetes. Diabetes Care. 2002;25(12):2335–41.

31. Mercer TH, Crawford C, Gleeson NP, Naish PF. Low-volume exercise rehabilitation improves functional capacity and self-reported functional status of dialysis patients. Am J Phys Med Rehabil [Internet]. 2002;81(3):162–7. Available from: http://www.ncbi.nlm.nih.gov/pubmed/11989511

32. Koufaki P, Mercer TH, Naish PF. Effects of exercise training on aerobic and functional capacity of end-stage renal disease patients. Clin Physiol Funct Imaging. 2002;22(2):115–24.

33. Mustata S, Groeneveld S, Davidson W, Ford G, Kiland K, Manns B. Effects of exercise training on physical impairment , arterial stiffness and health-related quality of life in patients with chronic kidney disease : a pilot study. Int Urol Nephrol. 2011;43(1):1133–41.

34. Viana JL, Kosmadakis GC, Watson EL, Bevington a., Feehally J, Bishop NC, et al. Evidence for Anti-Inflammatory Effects of Exercise in CKD. J Am Soc Nephrol [Internet]. 2014;25(9):2121–30. Available from: http://www.jasn.org/cgi/doi/10.1681/ASN.2013070702

35. Van Craenenbroeck AH, Van Ackeren K, Hoymans VY, Roeykens J, Verpooten G a., Vrints CJ, et al. Acute exercise-induced response of monocyte subtypes in chronic heart and renal failure. Mediators Inflamm. 2014;2014.

36. Johansen KL. Exercise and chronic kidney disease: current recommendations. Sport Med. 2005;35(6):485–99.

37. Singh SJ, Morgan MDL, Scott S, Walters D, Hardman AE. Development of a shuttle walking test of disability in patients with chronic airways obstruction. Thorax. 1992;47:1019–24.

38. Revill SM, Morgan MDL, Singh SJ, Williams J, Hardman a E. The endurance shuttle walk : a new field test for the assessment of endurance capacity in chronic obstructive pulmonary disease The endurance shuttle walk : a new field test for the assessment of endurance capacity in chronic obstructive pulmonary disease. Thorax. 1999;(July 2007):213–22.

39. Greenwood S a., Lindup H, Taylor K, Koufaki P, Rush R, MacDougall IC, et al. Evaluation of a pragmatic exercise rehabilitation programme in chronic kidney disease. Nephrol Dial Transplant. 2012;27(SUPPL. 3).

40. Wilund KR, Tomayko EJ, Wu P-T, Ryong Chung H, Vallurupalli S, Lakshminarayanan B, et al. Intradialytic exercise training reduces oxidative stress and epicardial fat: a pilot study. Nephrol Dial Transplant [Internet]. 2010;25(8):2695–701. Available from: http://ndt.oxfordjournals.org/cgi/doi/10.1093/ndt/gfq106

41. Painter P, Marcus RL. Assessing physical function and physical activity in patients with CKD. Clin J Am Soc Nephrol [Internet]. 2013;8(5):861–72. Available from: http://www.ncbi.nlm.nih.gov/pubmed/23220421

42. van der Heyde HC, Gramaglia I, Combes V, George TC, Grau GE. Flow Cytometric Analysis of Microparticles. Methods Mol Biol. 2011;699:337–54.

43. Ziegler-Heitbrock L, Hofer TPJ. Toward a Refined Definition of Monocyte Subsets. Front Immunol [Internet]. 2013;4(February):1–5. Available from: http://journal.frontiersin.org/article/10.3389/fimmu.2013.00023/abstract

For peer review only

For peer review only - http://bmjopen.bmj.com/site/about/guidelines.xhtml

44. Nielsen MH, Beck-Nielsen H, Andersen MN, Handberg A. A flow cytometric method for characterization of circulating cell-derived microparticles in plasma. J Extracell vesicles [Internet]. 2014;3:1–12. Available from: http://www.pubmedcentral.nih.gov/articlerender.fcgi?artid=3916676&tool=pmcentrez&rendertype=abstract

45. Jy W, Horstman L, Jiminez J, Ahn YS. Measuring circulating cell-derived microparticles. J Thromb Haemost. 2004;2(1):1842–51.

46. Eston R, Reilly T. Kinanthropometry and exercise physiology laboratory manual: anthropometry. 2009.

47. Chertow GM, Lowrie EG, Wilmore DW, Gonzalez J, Lew NL, Ling J, et al. Nutritional Assessment with Bioelectrical Impedance Analysis in Maintenance Hemodialysis Patients. J Am Soc Nephrol. 1995;6:75–81.

48. Squara P, Denjean D, Estagnasie P, Brusset A, Dib JC, Dubois C. Noninvasive cardiac output monitoring (NICOM): a clinical validation. Intensive Care Med. 2007;33:1191–4.

49. Obrador GT, Pereira BJG. Anaemia of chronic kidney disease : an under-recognized and under-treated problem. Nephrol Dial Transplant. 2002;177(S11):44–6.

50. Cheung WW, Paik KH, Mak RH. Inflammation and cachexia in chronic kidney disease. Pediatr Nephrol [Internet]. 2010;25(4):711–24. Available from: http://link.springer.com/10.1007/s00467-009-1427-z

51. Chao C-T, Huang J-W, Chiang C-K. Functional assessment of chronic illness therapy - the fatigue scale exhibits stronger associations with clinical parameters in chronic dialysis patients compared to other fatigue-assessing instruments. PeerJ. 2016;1818.

52. Jhamb M, Yabes J, Steel L, Amanda M, Unruh M. Prevalence and Correlates of Fatigue in Chronic Kidney Disease and End-Stage Renal Disease : Are Sleep Disorders a Key to Understanding Fatigue ? Am J Nephrol. 2013;38:489–95.

53. Gardner AW, Parker DE, Webb N, Montgomery PS, Scott KJ, Blevins SM, et al. Calf muscle hemoglobin oxygen saturation characteristics and exercise performance in patients with intermittent claudication. J Vasc Surg. 2008;48(3):644–9.

54. Wust RCI, Degens H. Factors contributing to muscle wasting and dysfunction in COPD patients. Int J COPD. 2007;2(3):289–300.

55. Grassi B, Quaresima V. Near-infrared spectroscopy and skeletal muscle oxidative function *in vivo* in health and disease: a review from an exercise physiology perspective. J Biomed Opt [Internet]. 2016;21(9):91313. Available from: http://biomedicaloptics.spiedigitallibrary.org/article.aspx?doi=10.1117/1.JBO.21.9.091313

For peer review only

For peer review only - http://bmjopen.bmj.com/site/about/guidelines.xhtml

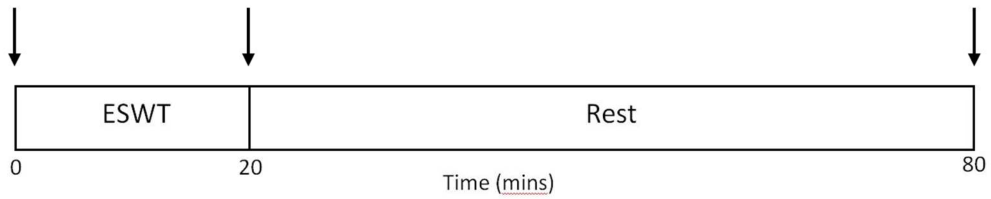

Figure 1. Visit 2 exercise and sample collection protocol. ↓= Venous blood and saliva collection. ESWT = Endurance Shuttle Walk Test

111x23mm (300 x 300 DPI)

For peer review only

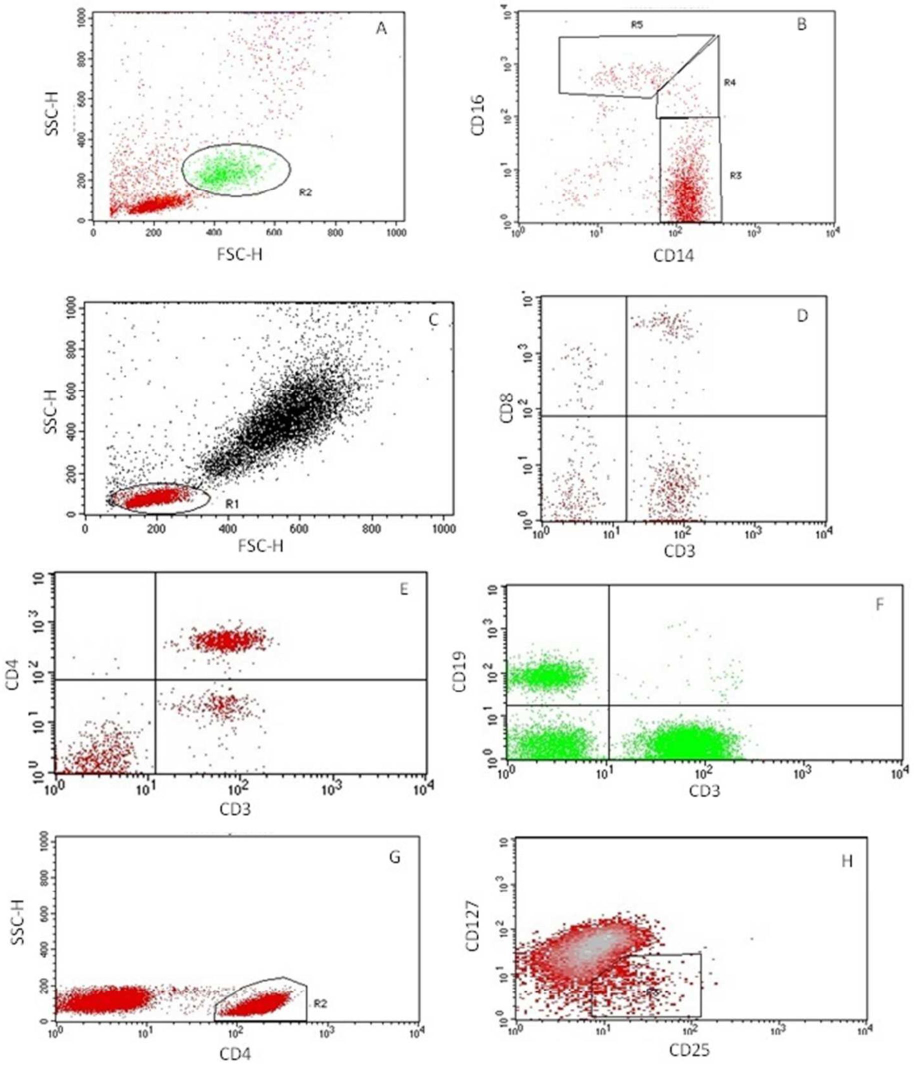

Figure 2. Demonstration of the gating strategy used to identify immune cell subsets. A: Total monocyte gating. B: Monocyte subsets (R3 = classical (CD14++CD16-), R4 = intermediate (CD14++CD16+), R5 = non-classical (CD14+CD16++) (R1 and R2 were used as preliminary gating to remove neutrophils). C: Total lymphocyte gating. D: Gating CD8+ cytotoxic T lymphocytes in the upper right quadrant. E: Gating CD4+ helper T lymphocytes in the upper right quadrant. F: Gating B lymphocytes (CD3-CD19+) in the upper left quadrant. G:Initial T-Reg gating, identifying CD4+ lymphocytes. H: Secondary T-reg gating, back-gated onto plot G, further identifying the CD4+ lymphocytes that are CD25+CD127-. Not all graphs display 100% of acquired cells – this has been altered independently to allow ease of gating.

64x70mm (300 x 300 DPI)

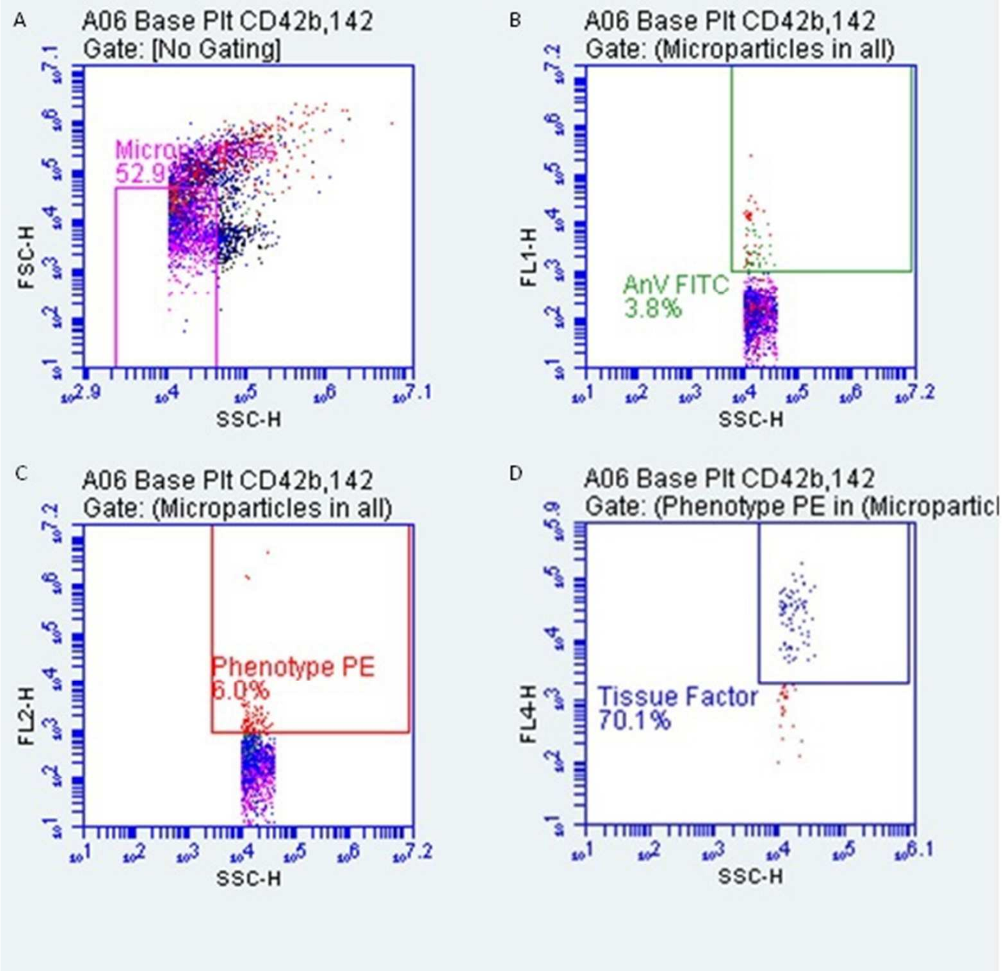

Figure 3. Demonstration of the gating strategy used to characterise microparticles. A: 'All microparticles', set using beads of known size. B: 'All microparticles' based on Annexin-v expression. C: Phenotype marker expression, used to quantify microparticles of different cellular sources. D: Pro-thrombotic potential, assessed by Tissue Factor expression. All gates have been appropriately sized and positioned using unstained samples to distinguish negative vs. positive staining.

52x51mm (300 x 300 DPI)

Participant Code :

Date :

Tiredness Scale

Place a mark on the horizontal line below after considering how you feel:

Generally over the last few days, how tired have you felt?

I have felt overwhelmingly tired and feel like I can't do anything |———————————| I haven't felt tired at all and I am full of energy

Over the last few days how tired have your muscles felt?

My muscles have felt very tired and heavy |———————————| My muscles haven't felt tired at all

Over the last few days how out of breath have you felt?

I have felt very out of breath |———————————| Not at all out of breath

Over the last few days how mentally tired or sleepy have you felt during the day?

I have felt very mentally tired or sleepy or have had trouble staying awake at times |———————————| I've felt wide awake and alert all day haven't felt sleepy at all

In your own words, describe how "being tired" feels to you?

LKET Fatigue Scale version 1 dated 3rd March 2014

For peer review only
